# Adult respondents’ perceptions of child abuse and formal child-protection institutions in Palestine and Jordan: a cross-sectional online survey

**DOI:** 10.3389/fpubh.2026.1859966

**Published:** 2026-07-21

**Authors:** Mohammad Amouri, Salahaldeen Deeb, Nour Sourkhi, Ruaa Issa, Osama Faqih, Maya Alothman, Razan Alnaimat, Lina Alja’afreh, Ahmad Thabet, Ahmad Raid Rabaiaa, Tariq Alhendi, Anas K. Assi

**Affiliations:** 1 Faculty of Medicine, Al-Quds University, Jerusalem, Palestine; 2 Department of Medicine, An-Najah National University, Nablus, Palestine; 3 School of Medicine, The University of Jordan, Amman, Jordan; 4 Faculty of Medicine, The Hashemite University, Zarqa, Jordan

**Keywords:** child abuse, child protection, institutional trust, Jordan, online survey, Palestine, reporting behavior

## Abstract

**Background:**

Public responses to child abuse depend on recognition of harm, knowledge of reporting pathways, perceived credibility of formal institutions, and broader social norms. This study examined these perceptions among adult online respondents in Palestine and Jordan.

**Methods:**

We conducted a cross-sectional online survey from 25 January to 12 April 2026 among adults residing in Palestine or Jordan. The Arabic questionnaire included 54 items covering sociodemographic characteristics and seven attitudinal domains: institutional trust and legitimacy, required staff expertise, perceived staff qualities, perpetrator accountability, institutional response orientation, media exposure, and punitive orientation. Composite scores were standardized to a 0–100 metric. Because “I do not know/cannot say for sure” responses were common for staff-quality items, uncertainty was analyzed descriptively and in sensitivity analyses.

**Results:**

The analytic sample included 779 respondents, 391 from Palestine and 388 from Jordan. The highest standardized means were observed for punitive orientation (73.35 ± 23.35) and required staff expertise (73.28 ± 19.62), whereas institutional trust and legitimacy remained below the midpoint (49.76 ± 17.24) and media exposure was lowest (39.58 ± 23.05). Staff-quality uncertainty was substantial: item-level uncertainty ranged from 30.4 to 39.0%, the mean number of uncertainty responses was 3.07 ± 3.37 across nine items, and 16.2% selected uncertainty for all nine items. Cross-country differences were generally small and were not significant after adjustment for measured sociodemographic covariates. Income was the most consistent measured correlate, but multivariable models explained modest variance (*R*^2^ range: 0.016–0.054). Sensitivity analyses for staff qualities did not materially change the main country-comparison findings.

**Conclusion:**

Among adult online respondents in Palestine and Jordan, endorsement of institutional involvement, multidisciplinary expertise, and perpetrator accountability exceeded confidence in institutions and procedural knowledge. These findings are not nationally representative.

## Introduction

Child abuse is among the most consequential yet least visible threats to child well-being, because its recognition is shaped not only by the occurrence of harm itself, but also by whether families, communities, and institutions define that harm as unacceptable and actionable. The World Health Organization defines child maltreatment as physical and emotional ill-treatment, sexual abuse, neglect, negligent treatment, and exploitation occurring in a relationship of responsibility, trust, or power, and emphasizes that its effects extend across the life course through injury, mental ill-health, impaired development, educational disadvantage, and wider social and economic costs (1). In this sense, child abuse is not merely a private family matter or a clinical problem; it is a public health, human rights, and governance issue whose persistence is closely tied to the capacity of societies to recognize abuse early and respond to it effectively through coordinated child protection systems (1, 2).

Recognition of abuse is deeply embedded in social norms. Practices that are framed within one community as discipline, family authority, or moral correction may be experienced by children as violence, humiliation, or coercion. Recent evidence shows that corporal punishment and other harmful disciplinary practices are often sustained by normative beliefs about obedience, parental entitlement, and the legitimacy of force in childrearing (3). At the same time, disclosure and reporting are socially patterned processes rather than simple individual choices. A recent systematic review of child sexual abuse disclosure found that disclosure is facilitated by supportive non-offending caregivers and inhibited by stigma, fear, relational dependency, and broader socio-cultural constraints (4). Similarly, a systematic review of reporting barriers among child-care professionals showed that uncertainty, fear of consequences, inadequate training, and low confidence in reporting pathways continue to impede formal reporting (5). Public knowledge also matters: higher levels of knowledge about child sexual abuse are associated with stronger support for preventive and policy responses, suggesting that awareness is not merely descriptive, but politically and institutionally consequential (6).

These dynamics place formal institutions at the center of child protection. Formal institutions include the legal, social welfare, health, and related administrative systems responsible for prevention, identification, reporting, referral, investigation, and protection. UNICEF’s child protection systems strengthening framework argues that effective child protection depends not only on legislation, but also on integrated services, trained personnel, intersectoral coordination, referral mechanisms, accountability structures, and community engagement (2). Just as importantly, institutional legitimacy matters. Comparative evidence indicates that public trust in child protection systems varies across jurisdictions and is shaped by institutional context, while trust itself is crucial for community cooperation and willingness to bring concerns to formal attention (7). Where institutions are perceived as inaccessible, ineffective, punitive, or socially distant, abuse may remain hidden even when communities are aware that harm has occurred (2, 7).

In the Arab region, however, the literature remains uneven and incomplete. A scoping review of child maltreatment and protection research in Gulf Cooperation Council countries concluded that the evidence base is still limited, with a strong concentration on descriptive and prevalence-oriented studies and insufficient attention to prevention, intervention, and system-level functioning (8). More recently, a systematic review across Arab countries found that physical, psychological, and sexual abuse have been studied far more often than neglect, reporting, and referral procedures, and explicitly highlighted the need for more research on reporting mechanisms and institutional pathways (9). This imbalance is important because child protection systems do not operate through legal texts alone; they depend on socially embedded judgments about whether abuse is recognizable, reportable, and appropriate for formal intervention. Yet comparative Arab research linking community awareness, cultural norms, institutional trust, and perceived institutional effectiveness remains scarce (8, 9).

Jordan provides a particularly relevant setting for examining these questions because it combines a relatively developed formal child-protection architecture with enduring social tolerance of violence against children. UNICEF Jordan notes that, despite a policy commitment to child protection, enforcement remains constrained by social and cultural norms, and violence in homes and other settings continues to be widely accepted (10). UNICEF also reports that nine out of ten children in Jordan experience violent discipline, underscoring the persistence of violence within everyday childrearing environments (10). At the same time, the National Study on Violence Against Children in Jordan documented extensive exposure to physical, psychological, and sexual violence and was explicitly designed to guide the further strengthening of the national child and family protection system (11). The adoption of Jordan’s Child Rights Law of 2022 further signaled a significant legal commitment to codifying children’s rights and clarifying the responsibilities of competent authorities, but legal reform does not automatically transform public attitudes, reporting practices, or confidence in institutions (12).

Palestine presents a distinct but equally urgent context. UNICEF State of Palestine describes the national child protection system as weak and nascent, marked by inadequate funding and capacity, fragmented jurisdiction, restricted access, uneven service reach, and low confidence in the formal social welfare sector, alongside continued reliance on informal dispute-resolution mechanisms (13). These structural constraints are compounded by broader political instability and social precarity. Empirical evidence from the West Bank has shown that child abuse is highly prevalent and socially patterned: Harsha and colleagues reported that approximately one third of children were exposed to high levels of abuse, with higher risk among boys, children of younger mothers, children whose fathers had lower educational attainment, and children whose families were exposed to political violence (14). In such a setting, community perceptions of abuse and institutional response are likely to be shaped not only by family norms, but also by lived experiences of fragmentation, insecurity, and differential access to services (13, 14).

Taken together, the literature points to four major gaps. First, research in Arab contexts has focused disproportionately on prevalence and type of abuse, while giving less attention to community interpretations of abuse, willingness to report, and evaluations of formal response systems (8, 9). Second, reporting behavior is often examined procedurally, rather than as a socially and culturally mediated process shaped by stigma, trust, perceived legitimacy, and expectations of institutional effectiveness (4, 5, 7). Third, there is limited comparative evidence from Arab countries that can illuminate how different sociopolitical and institutional environments shape public attitudes toward child protection. Fourth, although institutional trust is increasingly recognized as central to the operation of child protection systems, this construct remains underexamined in Middle Eastern settings specifically (7–9).

The present study addresses these gaps by examining perceptions of child abuse and formal institutional response among adult online respondents in Palestine and Jordan. The questionnaire measured seven domains: institutional trust and legitimacy, required staff expertise, perceived staff qualities, perpetrator accountability across five abuse scenarios, institutional response orientation, media exposure, and punitive orientation. We expected that institutional trust would be positively associated with more favorable ratings of staff qualities and stronger institutional response orientation, and we explored whether country and measured sociodemographic characteristics were associated with these observed domains. Because the questionnaire did not include a dedicated validated scale of general child-abuse awareness or normative acceptance of violent discipline, we do not frame the present analyses as direct tests of those constructs. These results should therefore be interpreted as evidence on the measured attitudinal domains in Palestine and Jordan rather than as broader claims about Arab populations.

## Methodology

### Study design and setting

We conducted a cross-sectional comparative online survey between 25 January 2026 and 12 April 2026 among adults residing in Palestine and Jordan. The questionnaire was self-administered electronically in Arabic using Google Forms. Participation was voluntary and uncompensated. Because recruitment relied on online dissemination rather than probability-based sampling, the study used a voluntary non-probability design. This approach was selected to facilitate anonymous participation on a socially sensitive topic and to reach eligible respondents in both settings; however, it remains susceptible to self-selection bias and likely overrepresentation of younger, more educated, and digitally connected individuals. The findings should therefore be interpreted as responses from this online sample and not as nationally representative estimates for either Palestine or Jordan.

### Participants

Eligible participants were adults aged 18 years or older who were residing in Palestine or Jordan, were able to read Arabic, and provided electronic informed consent before accessing the questionnaire. Individuals who did not provide consent were excluded automatically. A total of 780 responses were recorded during the study period; after exclusion of one non-consenting response, the final analytic sample comprised 779 respondents, including 391 from Palestine and 388 from Jordan. Because the survey link and QR code were disseminated through open online channels rather than through a closed sampling frame, a response rate could not be calculated.

### Recruitment and sampling

Participants were recruited through online dissemination using social media platforms and QR-code sharing. The survey link and QR code were circulated through commonly used online channels to reach eligible adults in Palestine and Jordan. Recruitment was not based on a probability sampling frame, and participation depended on voluntary self-selection after exposure to the survey link or QR code. To reduce duplicate submissions, the Google Forms setting requiring participants to sign in was enabled, and responses were restricted to one submission per Google account/email login. Duplicate-response prevention was therefore based on Google-account restriction rather than IP- or device-based blocking. No CAPTCHA or formal IP/device-based restriction was used. No rapid-response threshold was applied, and responses were not excluded on the basis of completion time. Although no dedicated bot-screening procedure was implemented, the risk of automated or duplicate submissions was minimized by requiring Google-account sign-in, limiting responses to one submission per account, and reviewing the dataset for incomplete or implausible entries during data cleaning.

### Questionnaire development and translation

The questionnaire used in this study was assembled by adapting item clusters from several previously published sources rather than by administering a single previously validated instrument. Items assessing trust in institutions dealing with child abuse, perceived staff qualities, perpetrator accountability, and media exposure were primarily adapted from the cross-national public-perception dataset reported by Mukai et al. (26). Items concerning reporting orientation and contact with formal institutions were informed by public-opinion work reported by LeCroy and Milligan-LeCroy and earlier public-attitudes studies. Wording relevant to child-abuse content in Arabic was additionally informed by the ICAST-R development literature (27), the Qatar–Palestine comparative adaptation of ICAST-R (28), and qualitative work from the West Bank emphasizing semantic clarity and cultural acceptability. Punitive-attitude items were informed by the harsher-punishment dimension of the Attitudes toward Criminal Justice Scale (29).

The final questionnaire contained 54 items organized into thematic sections covering sociodemographic characteristics and seven attitudinal domains. The questionnaire was translated into Arabic and then back-translated into English by an expert to support linguistic consistency and conceptual equivalence. The Arabic version was reviewed by two experts: one pediatric specialist and one Arabic/English language expert. Their feedback was used to improve clarity, linguistic accuracy, cultural appropriateness, and content validity before field administration. The questionnaire was then piloted among 30 participants before the main data collection. Pilot participants were not included in the final analytic sample. Feedback from the pilot phase was used to refine wording, formatting, and the estimated completion time. The consent form informed participants that the questionnaire would take approximately 5 min to complete, and this estimate was supported by pilot feedback. The full final questionnaire is provided as Supplementary material.

### Scoring and reliability

Responses were coded numerically so that higher scores reflected stronger endorsement of the construct being measured. Agreement-based items were coded from 1 to 6, importance items from 1 to 6, media-exposure items from 1 to 6, and responsibility-attribution items as binary indicators in which perpetrator attribution was coded as 1 and alternative attributions as 0. For staff-quality items, the response option “I do not know/cannot say for sure” was not treated as substantive endorsement of positive staff qualities and was excluded from the primary continuous score; however, because these responses were common, uncertainty was analyzed separately as a substantive outcome.

Composite scores were calculated for institutional trust and legitimacy, required staff expertise, perceived staff qualities, perpetrator accountability, institutional response orientation, media exposure, and punitive orientation, and then transformed to a standardized 0–100 metric. For the perceived staff qualities scale, the reported analytic sample was reproduced when at least five of the nine items were rated; this rule was therefore reported transparently in the revised analysis. Internal consistency was reported using Cronbach’s alpha with 95% confidence intervals, corrected item-total correlations, alpha-if-item-deleted values, and mean inter-item correlations. The perceived staff qualities scale showed extremely high internal consistency, which was interpreted cautiously as possibly reflecting semantic overlap or item redundancy rather than only strong reliability.

### Handling of uncertainty responses

For the perceived staff qualities items, the response option “I do not know/cannot say for sure” was treated as analytically distinct from negative evaluation. In the primary scoring approach, these responses were coded as missing for the continuous perceived staff qualities score because they indicated inability to judge institutional staff rather than low perceived staff quality. The perceived staff qualities score was calculated for respondents who provided valid ratings for at least five of the nine staff-quality items, which reproduced the reported analytic sample for this scale. Because uncertainty itself was conceptually meaningful, additional descriptive and sensitivity analyses were conducted. These included reporting the number and proportion of uncertainty responses for each staff-quality item, calculating the number of uncertainty responses per participant, identifying respondents who selected “I do not know/cannot say for sure” for all nine staff-quality items, comparing respondents included versus excluded from the primary perceived staff qualities score, and conducting alternative scoring analyses in which uncertainty responses were treated as a neutral midpoint.

### Data management and statistical analysis

Survey data were exported from Google Forms, checked for consent status, eligibility, coding consistency, completeness, and implausible entries, and then prepared for analysis. Duplicate prevention was implemented prospectively through the Google Forms sign-in requirement and one-response-per-Google-account setting. The survey did not use CAPTCHA, IP-based blocking, or device-based blocking. No rapid-response screening threshold was applied, and no response was excluded solely on the basis of completion time. Descriptive analyses summarized respondent characteristics and domain scores using frequencies, percentages, means, and standard deviations. Given the non-probability online design, these descriptive estimates are not interpreted as population-representative parameters.

Reliability analyses included Cronbach’s alpha with 95% confidence intervals, corrected item-total correlations, alpha-if-item-deleted statistics, and inter-item correlation summaries. Exploratory factor-analytic diagnostics were used to assess whether the domain structure was broadly compatible with the proposed scales. Because “I do not know/cannot say for sure” responses were concentrated in the staff-qualities domain, item-level uncertainty frequencies, participant-level uncertainty counts, and comparisons between respondents included versus excluded from the primary staff-quality score were also examined. Sensitivity analyses for that scale included stricter completeness thresholds and an alternative coding that assigned uncertainty responses to a neutral midpoint value.

Cross-country comparisons used chi-square tests for categorical variables and independent-samples *t* tests for continuous standardized outcomes. Multivariable linear regression models were fit for each standardized domain score with country as the principal comparative predictor and sex, age, educational level, income, and parenthood as covariates selected *a priori* because of their conceptual relevance and availability in the dataset. Model reporting included exact analytic N, unstandardized coefficients, standardized coefficients, 95% confidence intervals, *p* values, *R*^2^, adjusted *R*^2^, and variance inflation factors. Country-by-sociodemographic interaction terms were explored as secondary analyses. Because multiple outcomes and predictors were examined, *p* values were interpreted descriptively and the results were treated as exploratory.

### Ethical approval

The study protocol, questionnaire, and electronic consent procedure were reviewed and approved by the Research Ethics Committee of Al-Quds University and The University of Jordan. The study was conducted in accordance with the Declaration of Helsinki. Participation was voluntary and anonymous. Before entering the survey, participants were presented with an electronic information and consent form describing the study purpose, the expected survey duration, the sensitive nature of some questions, the absence of financial compensation, and the right to discontinue at any time before submission. Electronic informed consent was required before any questionnaire items were displayed. No direct identifiers, including names, telephone numbers, or national identification numbers, were collected. Data were analyzed and reported only in aggregate form.

## Results

### Sample characteristics

The analytic sample comprised 779 respondents, including 391 participants from Palestine and 388 from Jordan. In the pooled sample, the mean age was 29.60 ± 11.40 years, 583 respondents (74.8%) were aged 18–35 years, and women accounted for 445 participants (57.1%). The sample was predominantly highly educated, with 722 respondents (92.7%) reporting university education or higher. Single respondents constituted the largest marital-status group (499, 64.1%), students represented the largest employment subgroup (346, 44.4%), and most respondents reported average monthly income (465, 59.7%). Respondents with children represented 229 participants (29.4%). Detailed distributions are shown in Table 1.

**Table 1:** Sociodemographic characteristics overall and by country.

Variable	Category	Overall	Palestine	Jordan	Effect size	*p*-value
Age (years)	Mean ± SD	29.60 ± 11.40	28.08 ± 9.84	31.12 ± 12.62	*d* = −0.27	<0.001
Age group	18–35 years	583 (74.8)	314 (80.3)	269 (69.3)	V = 0.14	<0.001
	36–50 years	135 (17.3)	59 (15.1)	76 (19.6)		
	Over 50 years	59 (7.6)	17 (4.3)	42 (10.8)		
Sex	Male	334 (42.9)	188 (48.1)	146 (37.6)	V = 0.10	0.004
	Female	445 (57.1)	203 (51.9)	242 (62.4)		
Marital status	Single	499 (64.1)	253 (64.7)	246 (63.4)	V = 0.05	0.523
	Married	262 (33.6)	130 (33.2)	132 (34.0)		
	Divorced	14 (1.8)	5 (1.3)	9 (2.3)		
	Widowed	4 (0.5)	3 (0.8)	1 (0.3)		
Place of residence	City	587 (75.4)	256 (65.5)	331 (85.3)	V = 0.24	<0.001
	Village	169 (21.7)	123 (31.5)	46 (11.9)		
	Camp	16 (2.1)	9 (2.3)	7 (1.8)		
	Countryside	7 (0.9)	3 (0.8)	4 (1.0)		
Educational level	Preparatory	5 (0.6)	0 (0.0)	5 (1.3)	V = 0.11	0.012
	Secondary	52 (6.7)	33 (8.4)	19 (4.9)		
	University and above	722 (92.7)	358 (91.6)	364 (93.8)		
Employment status	Student	346 (44.4)	180 (46.0)	166 (42.8)	V = 0.19	<0.001
	Government employee	192 (24.6)	101 (25.8)	91 (23.5)		
	Private sector	95 (12.2)	36 (9.2)	59 (15.2)		
	Part-time work	39 (5.0)	27 (6.9)	12 (3.1)		
	Not working	47 (6.0)	21 (5.4)	26 (6.7)		
	Housewife	32 (4.1)	21 (5.4)	11 (2.8)		
	Retired	28 (3.6)	5 (1.3)	23 (5.9)		
Monthly income	Low	138 (17.7)	75 (19.2)	63 (16.2)	V = 0.05	0.423
	Average	465 (59.7)	225 (57.5)	240 (61.9)		
	High	176 (22.6)	91 (23.3)	85 (21.9)		
Has children	No	550 (70.6)	284 (72.6)	266 (68.6)	V = 0.04	0.242
	Yes	229 (29.4)	107 (27.4)	122 (31.4)		

Several sociodemographic characteristics differed between countries. Respondents in Jordan were older than those in Palestine (31.12 ± 12.62 vs. 28.08 ± 9.84 years, *t* = −3.74, *p* < 0.001; *d* = −0.27). The Jordanian subsample included a larger proportion of women (62.4% vs. 51.9%; χ^2^ = 8.27, *p* = 0.004) and a substantially higher proportion of city residents (85.3% vs. 65.5%; χ^2^ = 45.05, *p* < 0.001). By contrast, village residence was more common in Palestine (31.5% vs. 11.9%). Educational level and employment status also differed modestly by country, whereas marital status, income, and parenthood status did not show significant cross-country variation.

The sample profile indicates substantial selection toward younger, highly educated, digitally connected respondents. Nearly three quarters of respondents were aged 18–35 years, 92.7% reported university education or higher, and 44.4% were students. These features are important for interpretation because online recruitment is likely to underrepresent older adults, respondents with less formal education, those with limited internet access, and socially marginalized groups. The available dissemination approach did not provide a denominator for the number of adults who saw the survey link or QR code; therefore, a response rate could not be calculated.

### Scale reliability and overall descriptive performance

Internal consistency was acceptable to excellent for all multi-item scales in the pooled sample, with Cronbach’s *α* ranging from 0.743 for institutional trust and legitimacy to 0.925 for punitive orientation (Table 2). Country-specific reliability coefficients were closely comparable. The perceived staff qualities scale displayed exceptionally high internal consistency in complete-case analyses (*α* = 0.986 overall; 0.982 in Palestine and 0.989 in Jordan). These findings supported the use of the prespecified composite scores in both country-specific and pooled analyses.

**Table 2:** Reliability and descriptive statistics of the standardized scales.

Scale	Valid *n*	Cronbach *α* (overall)	Cronbach *α* (Palestine)	Cronbach *α* (Jordan)	RAW mean ± SD	0–100 mean ± SD	Palestine 0–100	Jordan 0–100	Mean difference (P − J)	95% CI	*p*-value	Effect size
Institutional trust and legitimacy	779	0.743	0.735	0.752	3.49 ± 0.86	49.76 ± 17.24	48.54 ± 16.84	50.98 ± 17.57	−2.44	−4.86 to −0.02	0.049	*d* = −0.14
Required staff expertise	779	0.919	0.903	0.932	4.66 ± 0.98	73.28 ± 19.62	73.17 ± 18.10	73.40 ± 21.06	−0.23	−3.00 to 2.53	0.868	*d* = −0.01
Perceived staff qualities	546	0.986	0.982	0.989	3.83 ± 1.19	56.61 ± 23.82	56.89 ± 22.74	56.34 ± 24.85	0.55	−3.45 to 4.55	0.787	*d* = 0.02
Perpetrator accountability	779	0.825	0.799	0.847	0.68 ± 0.36	67.88 ± 35.75	69.57 ± 34.19	66.19 ± 37.23	3.38	−1.65 to 8.41	0.187	*d* = 0.09
Institutional response orientation	779	0.836	0.811	0.859	3.82 ± 0.92	56.48 ± 18.43	55.77 ± 17.76	57.19 ± 19.07	−1.42	−4.01 to 1.17	0.283	*d* = −0.08
Media exposure	773	0.866	0.844	0.886	2.98 ± 1.15	39.58 ± 23.05	39.15 ± 21.53	40.01 ± 24.51	−0.86	−4.12 to 2.40	0.605	*d* = −0.04
Punitive orientation	778	0.925	0.916	0.933	4.67 ± 1.17	73.35 ± 23.35	73.79 ± 22.29	72.91 ± 24.41	0.88	−2.41 to 4.17	0.602	*d* = 0.04

**Figure 1:**
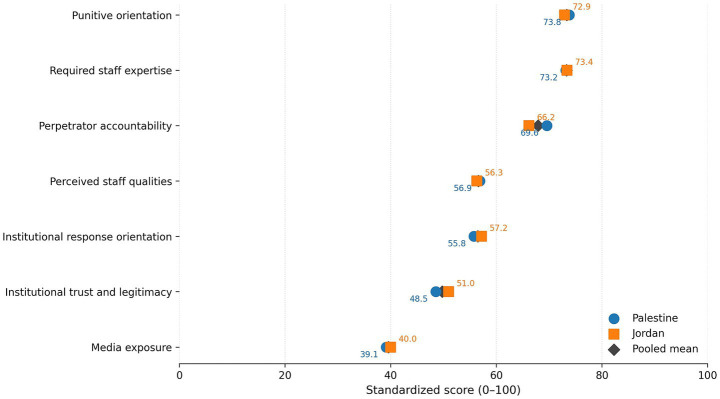
Standardized domain scores among adult online respondents in Palestine and Jordan. Points represent country means on the 0–100 standardized metric; the pooled mean is shown for reference.

On the 0–100 standardized metric, the highest mean scores were observed for punitive orientation (73.35 ± 23.35) and required staff expertise (73.28 ± 19.62), indicating strong endorsement of stricter sanctioning and strong expectations regarding staff qualifications. Perpetrator accountability was also relatively high (67.88 ± 35.75). Intermediate values were observed for perceived staff qualities (56.61 ± 23.82) and institutional response orientation (56.48 ± 18.43), whereas institutional trust and legitimacy remained below the midpoint (49.76 ± 17.24). Media exposure produced the lowest overall score (39.58 ± 23.05). Figure 1 shows that the overall scale profile was highly similar in Palestine and Jordan, with only a small difference in institutional trust and legitimacy.

A distinctive feature of the dataset was the extent of uncertainty surrounding perceived staff qualities. After application of the prespecified missing-data rule for “I do not know / cannot say for sure” responses, 233 respondents (29.9%) did not meet the minimum threshold for calculation of the perceived staff qualities score. The mean number of uncertainty responses across the nine staff-quality items was 3.07 ± 3.37, and 126 respondents (16.2%) selected the uncertainty option for all nine items. This level of uncertainty was considerably greater than the small amount of missingness observed for the other scales.

Uncertainty regarding institutional staff qualities appeared to be distributed broadly rather than being strongly concentrated within a single measured subgroup. Across the nine staff-quality items, uncertainty frequencies ranged from 30.4 to 39.0%. The mean number of uncertainty responses was 3.07 ± 3.37 items per respondent, and 16.2% of respondents selected the uncertainty option for all nine items. Comparisons between respondents included versus excluded from the primary staff-quality score did not show strong evidence of systematic differences by country, sex, education, income, employment, residence, marital status, or parenthood, and age-group differences were at most borderline. These findings suggest that limited visibility of institutional staff may be a broadly shared feature of the sample rather than a phenomenon confined to one measured subgroup.

Psychometric reporting was expanded beyond Cronbach’s alpha alone. Internal consistency was acceptable to excellent for the multi-item scales, with Cronbach’s alpha values of 0.743 for institutional trust and legitimacy, 0.919 for required staff expertise, 0.825 for perpetrator accountability, 0.836 for institutional response orientation, 0.866 for media exposure, and 0.925 for punitive orientation. The perceived staff qualities scale was exceptionally high (*α* = 0.986; 95% CI approximately 0.982–0.989), with very high corrected item-total correlations and mean inter-item correlation, indicating a strongly overlapping item set. Exploratory dimensionality diagnostics supported a dominant one-factor solution for most scales. However, institutional response orientation showed evidence of a possible two-component structure, with normative/pro-intervention items clustering separately from self-reported knowledge items; this domain should therefore be interpreted as a composite rather than as a fully validated unidimensional scale.

### Cross-country scale and item profiles

At the composite-score level, cross-country differences were limited. Jordan scored slightly higher than Palestine on institutional trust and legitimacy (50.98 ± 17.57 vs. 48.54 ± 16.84), corresponding to a small mean difference of 2.44 points (95% CI, 0.02 to 4.86; *p* = 0.049; *d* = 0.14 in absolute value). No other standardized scale differed significantly between countries, and all remaining effect sizes were negligible to small (|*d*| ≤ 0.09; Table 2).

Item-level inspection showed that the weakest domain in both countries was direct confidence in institutions. Within the institutional trust and legitimacy scale, emotional closeness to public institutions yielded the highest pooled item mean (55.28/100), followed by perceived professionalism (50.55/100), whereas confidence in institutions was the lowest-scoring item (45.88/100). Among expertise items, respondents most strongly endorsed psychology (79.85/100), social welfare (79.79/100), and education and training (78.36/100), whereas economics was clearly the least endorsed expertise area (55.92/100). For perceived staff qualities, the most favorably rated attributes were friendliness (60.30/100), kind-heartedness (59.65/100), and collaboration (59.15/100), whereas intelligence, skill, and competence occupied the lower end of the observed range.

Institutional response orientation was characterized by strong normative support for engaging formal institutions but weaker self-reported knowledge. The highest-scoring items in this domain were the belief that contacting a formal institution is in the child’s best interest (66.57/100) and willingness to contact an institution if abuse were suspected (63.80/100). By contrast, respondents reported much lower knowledge of the relevant institutions (43.08/100) and of how to respond appropriately to a child if abuse were suspected (50.78/100). Media exposure remained modest, with the highest exposure reported for social media and online news, and the lowest exposure for newspapers. Punitive orientation items were uniformly high, with pooled means ranging from 66.10 to 76.56/100.

Top-box endorsement patterns reinforced these item-level gradients. In the institutional trust domain, only 11.2 to 27.9% of respondents selected the two most favorable response categories, with the highest endorsement again observed for emotional closeness and the lowest for perceived social status. By contrast, 51.2 to 69.1% of respondents selected the upper two categories for the expertise items, and 48.0 to 63.9% did so for punitive-orientation items. Endorsement for positive staff qualities was more restrained, ranging from 26.7 to 38.6%, further underscoring the combination of strong normative expectations for institutions and comparatively cautious evaluations of their personnel.

Only three item-level cross-country differences reached statistical significance. Jordan reported higher confidence in institutions than Palestine (49.90 vs. 41.89, *p* < 0.001), higher perceived knowledge of relevant institutions (45.10 vs. 41.07, *p* = 0.032), and slightly greater exposure through newspapers (28.50 vs. 24.79, *p* = 0.041). No other item-level differences between countries were statistically significant; full item-level cross-country comparisons are provided in Supplementary Table S9.

Attribution of responsibility for child abuse showed a consistent pattern across both settings. Perpetrators were the most frequently selected responsible party in all five scenarios, ranging from 61.5% for exposing children to domestic violence to 70.9% for physical abuse (Table 3). Public institutions were selected by 9.9 to 14.9% of respondents, society by 11.2 to 17.5%, and other parties by 4.6 to 7.2%. The lowest perpetrator attribution occurred for exposure to domestic violence, which also generated the highest proportion of society responses. Cross-country differences in the full four-category attribution distribution were small. Only physical abuse showed a statistically significant country difference (χ^2^ = 9.46, *p* = 0.024, Cramér’s V = 0.11), with slightly higher perpetrator attribution in Palestine than Jordan (72.1% vs. 69.6%). The remaining scenarios did not differ significantly between countries.

**Table 3:** Attribution of primary responsibility for child abuse scenarios.

Abuse scenario	Perpetrators, overall *n* (%)	Public institutions, overall *n* (%)	Society, overall *n* (%)	Other parties, overall *n* (%)	Palestine perpetrators *n* (%)	Jordan perpetrators *n* (%)	χ^2^	*p*-value	Cramér’s V
Physical abuse	552 (70.9)	104 (13.4)	87 (11.2)	36 (4.6)	282 (72.1)	270 (69.6)	9.46	0.024	0.11
Psychological abuse	551 (70.7)	77 (9.9)	112 (14.4)	39 (5.0)	282 (72.1)	269 (69.3)	3.04	0.385	0.06
Sexual abuse	520 (66.8)	111 (14.2)	92 (11.8)	56 (7.2)	266 (68.0)	254 (65.5)	6.52	0.089	0.09
Neglect	542 (69.6)	94 (12.1)	97 (12.5)	46 (5.9)	284 (72.6)	258 (66.5)	3.95	0.267	0.07
Exposure to domestic violence	479 (61.5)	116 (14.9)	136 (17.5)	48 (6.2)	246 (62.9)	233 (60.1)	6.51	0.089	0.09

### Sociodemographic comparisons

Bivariate subgroup comparisons revealed little heterogeneity by country, sex, or parenthood for most outcomes (Table 4). Detailed subgroup comparisons by age group, residence type, and income are provided in Supplementary Table S10. Women reported slightly stronger support for required staff expertise than men (74.79 ± 18.74 vs. 71.27 ± 20.59; *p* = 0.014; *d* = 0.18) and slightly higher institutional response orientation (57.65 ± 17.86 vs. 54.92 ± 19.07; *p* = 0.043; *d* = 0.15). Participants with children expressed somewhat stronger punitive orientation than those without children (75.87 ± 22.61 vs. 72.31 ± 23.60; *p* = 0.049; *d* = 0.15). No pooled differences by residence type reached significance for any standardized scale. The full bivariate subgroup results by age group, residence type, and income are presented in Supplementary Table S10.

**Table 4:** Standardized scale scores according to country, sex, and parental status.

Scale	Palestine mean ± SD	Jordan mean ± SD	*p* (country)	*d* (country)	Male mean ± SD	Female mean ± SD	*p* (sex)	*d* (sex)	No children mean ± SD	Has children mean ± SD	*p* (children)	*d* (children)
Institutional trust and legitimacy	48.54 ± 16.84	50.98 ± 17.57	0.049	−0.14	48.40 ± 17.83	50.78 ± 16.73	0.059	−0.14	49.74 ± 17.12	49.80 ± 17.56	0.965	−0.00
Required staff expertise	73.17 ± 18.10	73.40 ± 21.06	0.868	−0.01	71.27 ± 20.59	74.79 ± 18.74	0.014	−0.18	72.97 ± 19.97	74.05 ± 18.75	0.472	−0.06
Perceived staff qualities	56.89 ± 22.74	56.34 ± 24.85	0.787	0.02	56.02 ± 24.14	57.06 ± 23.60	0.613	−0.04	57.01 ± 24.16	55.59 ± 22.98	0.523	0.06
Perpetrator accountability	69.57 ± 34.19	66.19 ± 37.23	0.187	0.09	67.90 ± 36.21	67.87 ± 35.44	0.988	0.00	66.73 ± 35.86	70.66 ± 35.42	0.161	−0.11
Institutional response orientation	55.77 ± 17.76	57.19 ± 19.07	0.283	−0.08	54.92 ± 19.07	57.65 ± 17.86	0.043	−0.15	56.02 ± 18.49	57.57 ± 18.28	0.285	−0.08
Media exposure	39.15 ± 21.53	40.01 ± 24.51	0.605	−0.04	38.02 ± 22.48	40.75 ± 23.43	0.100	−0.12	39.72 ± 23.61	39.24 ± 21.69	0.786	0.02
Punitive orientation	73.79 ± 22.29	72.91 ± 24.41	0.602	0.04	71.63 ± 24.18	74.64 ± 22.66	0.078	−0.13	72.31 ± 23.60	75.87 ± 22.61	0.049	−0.15

Age-group differences were also limited. The only significant age effect was observed for perceived staff qualities (*F* = 4.47, *p* = 0.012, η^2^ = 0.016), with the lowest scores among respondents aged over 50 years (46.87 ± 18.61). *Post hoc* testing indicated that the over-50 group scored significantly lower than the 18–35-year group (Tukey-adjusted *p* = 0.009), whereas the remaining pairwise contrasts were not significant. No age-group differences were observed for institutional trust and legitimacy, required staff expertise, perpetrator accountability, institutional response orientation, media exposure, or punitive orientation.

**Figure 2:**
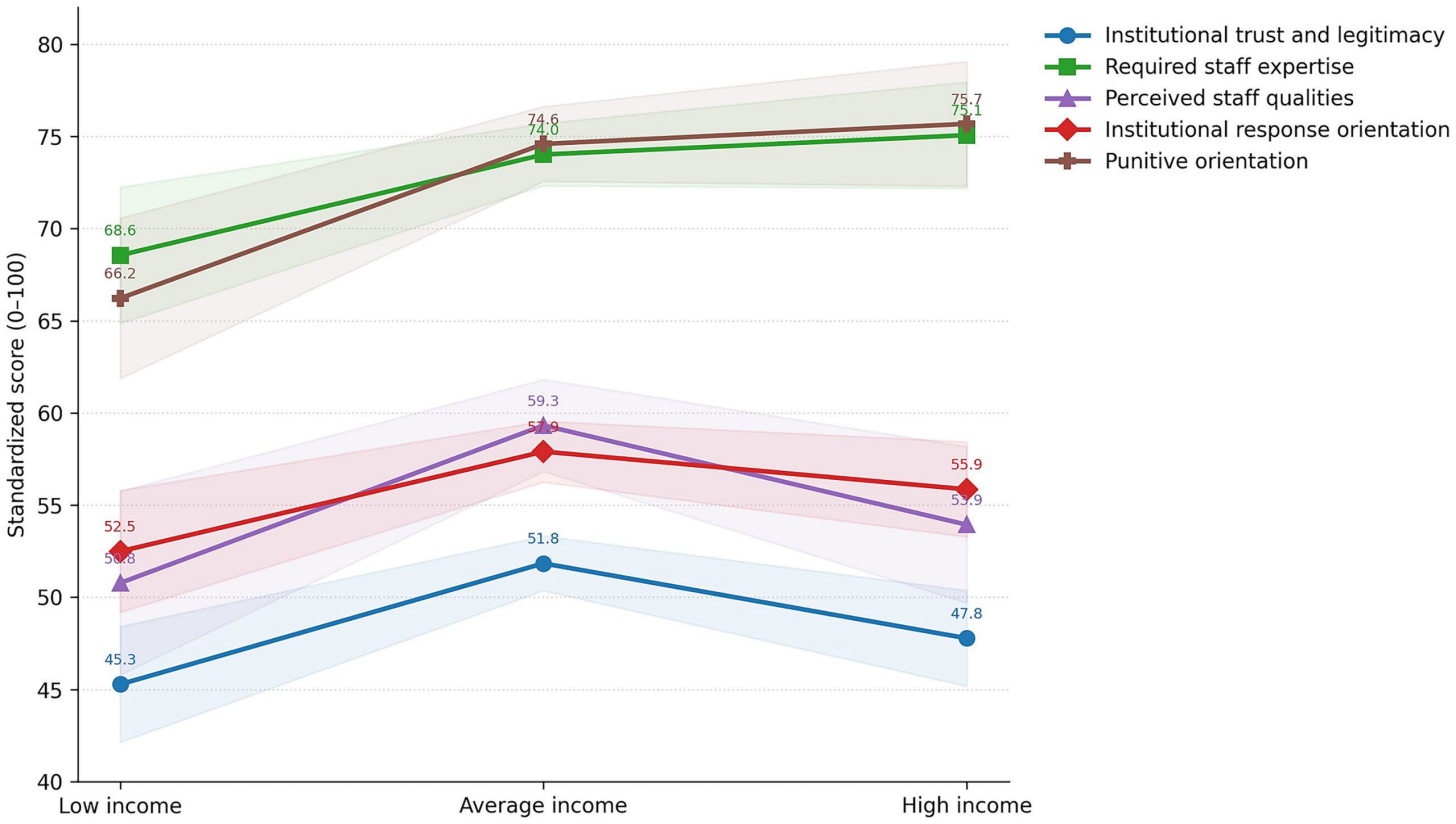
Mean standardized scores across self-reported income groups among adult online respondents in Palestine and Jordan.

Income represented the clearest and most consistent source of subgroup variation. Institutional trust and legitimacy differed across income groups (*F* = 9.41, *p* < 0.001, η^2^ = 0.024), with the highest mean observed in the average-income group (51.84 ± 16.29) and the lowest in the low-income group (45.28 ± 18.82). Required staff expertise also varied by income (*F* = 5.12, *p* = 0.006, η^2^ = 0.013), with both average- and high-income respondents scoring higher than low-income respondents in *post hoc* comparisons. Perceived staff qualities (*F* = 5.85, *p* = 0.003, η^2^ = 0.021), institutional response orientation (*F* = 4.77, *p* = 0.009, η^2^ = 0.012), media exposure (*F* = 4.76, *p* = 0.009, η^2^ = 0.012), and punitive orientation (*F* = 8.11, *p* < 0.001, η^2^ = 0.021) also differed significantly by income. The average-income group scored higher than the low-income group on institutional trust, perceived staff qualities, and institutional response orientation, whereas both average- and high-income groups exceeded the low-income group on punitive orientation. Media exposure showed a slightly different pattern, with average-income respondents scoring higher than high-income respondents (Figure 2).

### Correlations and multivariable associations

**Figure 3:**
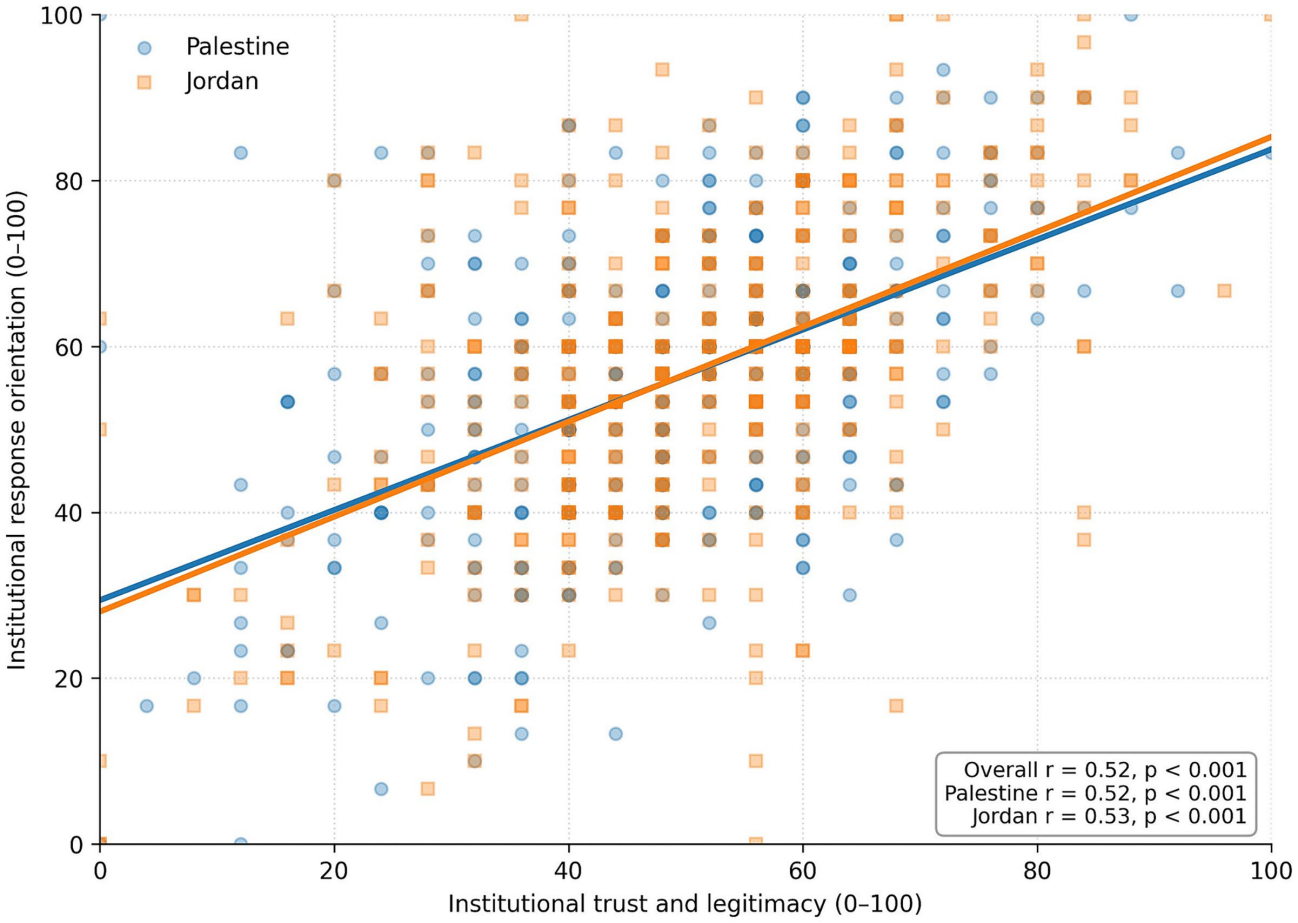
Association between institutional trust and institutional response orientation among respondents with valid scores, shown overall and separately for Palestine and Jordan.

All standardized scales were positively intercorrelated, although the magnitude of these associations varied. The strongest correlations were observed between institutional trust and perceived staff qualities (*r* = 0.58, *p* < 0.001), perceived staff qualities and institutional response orientation (*r* = 0.58, *p* < 0.001), required staff expertise and punitive orientation (*r* = 0.57, *p* < 0.001), and institutional trust and institutional response orientation (*r* = 0.52, *p* < 0.001). The association between institutional trust and institutional response orientation was evident in both Palestine (*r* = 0.52, *p* < 0.001) and Jordan (*r* = 0.53, *p* < 0.001), as illustrated in Figure 3. By contrast, perpetrator accountability showed only weak associations with the remaining scales (*r* values approximately 0.10–0.21), and media exposure was modestly related to institutional response orientation (*r* = 0.33, *p* < 0.001).

Adjusted linear regression models confirmed that the crude cross-country difference in institutional trust and legitimacy was modest and did not remain significant after controlling for sex, age, education, income, and parenthood (Jordan vs. Palestine: B = 2.06, 95% CI, −0.38 to 4.49; *p* = 0.098). Income remained the most consistent multivariable correlate across outcomes. Full multivariable regression models, including model-specific *N* values, unstandardized coefficients, standardized coefficients, 95% confidence intervals, *p* values, *R*^2^, adjusted *R*^2^, and variance inflation factors, are provided in Supplementary Table S7. Compared with low income, average income was independently associated with higher institutional trust and legitimacy (B = 6.51, 95% CI, 3.23 to 9.80; *p* < 0.001), required staff expertise (B = 5.67, 95% CI, 1.92 to 9.43; *p* = 0.003), perceived staff qualities (B = 9.86, 95% CI, 4.46 to 15.27; *p* < 0.001), institutional response orientation (B = 5.53, 95% CI, 2.00 to 9.06; *p* = 0.002), and punitive orientation (B = 8.38, 95% CI, 3.92 to 12.85; *p* < 0.001). High income was independently associated with higher required staff expertise (B = 6.81, 95% CI, 2.40 to 11.22; *p* = 0.003) and punitive orientation (B = 9.79, 95% CI, 4.55 to 15.03; *p* < 0.001).

Additional adjusted associations were more selective. Female respondents reported higher required staff expertise than male respondents (B = 3.26, 95% CI, 0.45 to 6.08; *p* = 0.023), while older age was associated with lower required staff expertise (B = −0.19 per year, 95% CI, −0.35 to −0.03; *p* = 0.020) and lower perceived staff qualities (B = −0.42 per year, 95% CI, −0.64 to −0.20; *p* < 0.001). University education or higher was associated with greater perpetrator accountability relative to secondary education or lower (B = 13.83, 95% CI, 3.94 to 23.73; *p* = 0.006). Having children was independently associated with higher punitive orientation (B = 4.81, 95% CI, 0.10 to 9.52; *p* = 0.045). The proportion of explained variance was modest across models (*R*^2^ range, 0.016–0.054), with the largest value observed for perceived staff qualities.

Sensitivity analyses for the perceived staff qualities domain did not materially alter the main comparative interpretation. Using the primary scoring rule reproduced the originally reported scored sample (*n* = 546). Stricter completeness thresholds reduced the scored sample, as expected, but did not introduce meaningful Palestine–Jordan differences. An alternative analysis that coded uncertainty responses to a neutral midpoint lowered the overall mean score but likewise left the country comparison essentially unchanged. Correlations of perceived staff qualities with institutional trust and institutional response orientation remained moderate to strong across all coding strategies. These analyses support the substantive conclusion that the central issue is not a hidden country difference in staff evaluations, but rather the unusually high prevalence of uncertainty itself.

## Discussion

The revised interpretation of this study should begin from two facts: first, the sample consisted of voluntary adult online respondents rather than a representative population sample; and second, the measured associations explained only a modest proportion of variance. Within those limits, the data suggest that respondents in both Palestine and Jordan showed stronger endorsement of punitive responses, multidisciplinary staff expertise, perpetrator accountability, and formal institutional involvement than of institutional trust, procedural knowledge, or exposure to child-protection information. In other words, support for institutional involvement appeared stronger than confidence in institutions or familiarity with institutional processes.

This pattern may suggest a gap between normative endorsement of child protection and practical confidence in formal response systems, but that interpretation should remain cautious. Constructs such as “institutional readiness,” “public legitimacy,” and “relational distance” were not measured directly in this survey. Rather, they are theoretical lenses through which the observed combination of lower trust, lower procedural knowledge, low media exposure, and high uncertainty about staff qualities may be interpreted. The discussion therefore distinguishes between direct empirical findings and broader interpretive framing.

The findings on perpetrator accountability further illuminate the normative architecture of public perceptions. Across both settings, perpetrators were the most frequently identified responsible party in all abuse scenarios, indicating that respondents generally rejected narratives that excuse abuse or shift primary responsibility away from offenders. This is an encouraging finding because it suggests that public moral reasoning is broadly aligned with rights-based child protection principles. However, the comparatively lower perpetrator attribution for children’s exposure to domestic violence, together with the greater tendency to assign responsibility to society in that scenario, indicates that some forms of harm remain more socially diffused than others. This may reflect the continued embedding of violence within broader understandings of family life, social conflict, and relational order. Such an interpretation is consistent with evidence showing that corporal punishment and other harmful disciplinary practices are maintained by powerful norms concerning obedience, parental authority, and the legitimacy of force in childrearing (3). It also aligns with qualitative evidence from the West Bank indicating that child abuse is discussed within a culturally sensitive terrain shaped by social silence, semantic ambiguity, and concern about acceptability and family reputation (15). In this sense, the present study suggests that while many respondents clearly identify overt abuse as a wrongdoing attributable to perpetrators, more relational or family-embedded forms of harm may still be interpreted through wider social and normative frameworks.

The low media exposure observed in both countries is another notable finding. Public communication is a crucial mechanism through which abuse becomes socially visible, reporting becomes imaginable, and institutions become recognizable. In the present study, media exposure was the weakest overall domain, with modest engagement even through online and social media sources. This has important implications because awareness campaigns and media discourse can shape both knowledge and perceived legitimacy. Public-opinion work has long shown that attitudes toward child maltreatment are influenced by the ways in which abuse is framed and discussed socially (16–18). More recent work suggests that public understanding of abuse is linked to support for preventive policies (6), while reviews of universal prevention campaigns indicate that media-based approaches can improve knowledge, self-efficacy, and certain prevention-related attitudes, even when evidence for behavior change remains mixed (19). In social contexts where shame, stigma, and the preservation of family reputation may inhibit open discussion of abuse, weak exposure to child-protection content may reinforce uncertainty and passivity rather than mobilize reporting. The current findings therefore suggest that child protection remains insufficiently institutionalized in the everyday public information environment of both Palestine and Jordan.

The institutional trust findings are especially revealing. Although respondents supported formal intervention, institutional trust and legitimacy remained below the midpoint, and direct confidence in institutions was one of the weakest item-level indicators. This suggests that respondents endorse the principle of institutional involvement more strongly than they trust the institutions currently responsible for carrying it out. That distinction is highly consequential. Trust is a central condition for public cooperation with formal institutions, particularly in domains involving vulnerability, discretion, and stigma. Comparative work has shown that trust in child protection systems varies significantly across institutional contexts and is shaped by citizens’ perceptions of competence, fairness, and effectiveness (7, 20). Hassan’s comparative analysis of England and Norway further demonstrates that procedural fairness and perceived competence are key determinants of trust in child protection systems (20). More broadly, welfare-state scholarship has emphasized that citizens’ trust in institutions depends not only on formal design, but also on lived experiences of bureaucratic treatment, frontline interactions, and perceptions of justice (21). Against this backdrop, the present findings suggest that support for child protection in Palestine and Jordan is constrained not by wholesale rejection of formal systems, but by limited confidence in their practical credibility.

This interpretation is reinforced by the unusually high level of uncertainty surrounding perceived staff qualities. Nearly one third of respondents did not meet the threshold for calculation of the staff-quality scale because they too frequently selected “I do not know/cannot say for sure,” and a substantial subgroup selected that response for all nine items. This is not merely a psychometric issue; it is substantively important. It implies that, for many respondents, institutional personnel are not socially visible enough to be judged confidently. In other words, the system may be known in abstract terms but not embodied in recognizable, trusted actors. This matters because institutions are often encountered and evaluated through their representatives. The strong correlations in the present study between perceived staff qualities, institutional trust, and institutional response orientation suggest that trust is relational as much as structural. This interpretation is consistent with procedural justice theory, which emphasizes that legitimacy depends not only on outcomes, but on perceptions of respectful, fair, competent, and trustworthy conduct by authorities (22). Thus, one of the core barriers to stronger institutional legitimacy in these settings may be the relational distance between child protection systems and the communities they are meant to serve.

At the same time, respondents expressed very strong support for multidisciplinary expertise, especially in psychology, social welfare, and education or training. This is a notable strength in the public perception profile because it suggests that abuse is not understood solely as a criminal-justice matter. Rather, respondents appear to recognize child protection as a complex, multidisciplinary field requiring psychosocial, legal, educational, and health-related competence. This aligns closely with systems-strengthening frameworks, which emphasize intersectoral coordination, trained personnel, referral pathways, and integrated service delivery (2). Yet this support for expertise coexisted with very strong punitive orientation. Respondents did not choose between welfare and punishment; they endorsed both. This coexistence is important because it suggests that communities expect child protection systems simultaneously to care, protect, discipline, and sanction. Such a pattern may reflect a demand for visible decisiveness where trust remains incomplete. It may also indicate that the public imagines institutional effectiveness partly through the lens of punitive accountability, especially in contexts where abuse is morally condemned but service systems are not fully trusted.

The relatively small differences between Palestine and Jordan are among the most theoretically significant findings of the study. Jordan showed only a slight advantage in institutional trust and knowledge of relevant institutions, and this country effect did not persist in adjusted models. At one level, this result is unsurprising. Jordan has a comparatively more consolidated formal child-protection architecture, has conducted a national study on violence against children, and has enacted the Child Rights Law of 2022 (10–12). Palestine, by contrast, has been described as having a more fragmented and under-resourced child protection system operating under conditions of political instability and territorial fragmentation (13). However, the modest scale of the observed country difference suggests that institutional architecture alone does not determine public perceptions. Shared regional norms around family privacy, child discipline, stigma, and authority may narrow the extent to which legal and policy differences translate into sharply divergent public attitudes. This interpretation is consistent with regional reviews showing that Arab child protection research remains skewed toward prevalence rather than institutional functioning, reporting, and legitimacy (8, 9). In short, the present findings suggest that, among these adult online respondents, attitudes toward child protection may be shaped by shared social norms as well as by differences in national policy infrastructure.

Income, rather than nationality, emerged as the clearest and most consistent source of variation. Compared with low-income respondents, those in the average-income group reported higher institutional trust, stronger support for staff expertise, more positive perceptions of staff qualities, greater institutional response orientation, and stronger punitive orientation, while both average- and high-income respondents exceeded the low-income group on several outcomes. This is an important finding because it suggests that socioeconomic position shapes not only exposure to vulnerability, but also how institutions are imagined, judged, and considered usable. The socioecological literature has repeatedly shown that child maltreatment is embedded in broader conditions of social and material hardship (23). More recently, economic and concrete support has been reframed as a central preventive strategy rather than a secondary adjunct to child protection (24). The present findings extend that logic from risk production to public perception. Material position appears to influence whether institutions are seen as trustworthy, whether intervention is endorsed, and whether protective systems appear meaningful. Particularly striking is the contrast between explicit attitudes and structural patterning: economics was the least endorsed area of needed staff expertise, yet income was the strongest and most consistent correlate across outcomes. This may indicate that respondents continue to conceptualize child abuse primarily in moral, interpersonal, and disciplinary terms while underestimating the structural and material conditions that shape vulnerability, disclosure, and institutional confidence.

The findings also have important implications for theory. First, they support a social-ecological interpretation of child protection attitudes. The study suggests that perceptions of abuse, reporting, and institutional legitimacy are shaped not only by individual beliefs, but also by relational, institutional, and structural factors such as media visibility, social norms, income, and the perceived accessibility of services (23). Second, the findings contribute to institutional trust theory by showing that trust in child protection is not a secondary attitudinal variable; it is central to whether the public regards formal systems as meaningful agents of response (7, 20, 21). Third, the coexistence of high punitive orientation and high support for multidisciplinary institutional roles highlights a tension between retributive public sentiment and the more preventive, therapeutic, and welfare-oriented logic of many child-protection systems. Procedural justice approaches may be especially useful here because they help explain how communities can simultaneously demand strong accountability and still evaluate institutions through relational qualities such as fairness, competence, and respect (22). For Palestine and Jordan, this may imply that strengthening perceived child-protection legitimacy requires both more visible accountability and more humane, comprehensible, and trusted service delivery.

The practical implications are immediate. First, awareness efforts in both countries should move beyond general messages condemning child abuse and instead provide clear, repeated, and practical information on where to report, what happens after reporting, which institutions are responsible, and what protections or services families and children can expect. The present study indicates that broad moral support already exists; what is weaker is procedural knowledge. Second, formal systems require greater public visibility. The uncertainty surrounding staff qualities suggests that many respondents do not know enough about institutional personnel to evaluate them. Public-facing communication, transparent referral pathways, community-based outreach, and visible multidisciplinary teams may therefore be as important as internal institutional reform. Third, training remains important. Evidence from a Cochrane review suggests that child-protection training may improve knowledge and some reporting-related outcomes among professionals, even though the certainty of evidence remains limited (25). Fourth, reforms should avoid equating child protection solely with punishment. Although respondents strongly supported harsher sanctions, they also valued psychosocial and educational expertise. Effective reform must therefore combine credible accountability with therapeutic, preventive, and family-supportive responses when safe and appropriate (2). Finally, the strong income gradient indicates that child protection policy should be integrated with broader social support and anti-poverty strategies, especially for economically vulnerable families (24).

### Social desirability bias

The possibility of social desirability bias also deserves stronger emphasis. High scores on punitive orientation, strong perpetrator accountability, and reported willingness to contact institutions may partly reflect socially normative responding on a sensitive topic rather than actual behavioral readiness. Self-reported endorsement of reporting or institutional involvement should not be assumed to translate directly into real-world reporting behavior when abuse is encountered. Future research would benefit from vignette-based designs, qualitative interviewing, behavioral-intention measures, and, where feasible, longitudinal or mixed-method approaches that can better distinguish publicly endorsed norms from enacted practice.

### Limitations

Several limitations should be acknowledged. First, the study used a voluntary online non-probability sample that was heavily skewed toward younger, highly educated, and digitally connected respondents; accordingly, the findings should not be interpreted as nationally representative of Palestine or Jordan. Second, although duplicate submissions were minimized by requiring Google-account sign-in and restricting responses to one submission per account, the survey did not use CAPTCHA or formal IP/device-based blocking, and no rapid-response threshold was applied. Third, although the questionnaire underwent translation, back-translation, expert review, and pilot testing among 30 participants, it was adapted from multiple sources rather than administered as a single previously validated instrument. Therefore, construct validity should be interpreted cautiously and further psychometric evaluation is needed in future representative samples. Fourth, although the multi-item domains were internally consistent, the extremely high internal consistency of the perceived staff qualities scale suggests substantial item overlap, and the institutional response orientation scale may contain both normative-support and procedural-knowledge subdimensions. Fifth, “I do not know/cannot say for sure” responses were highly prevalent for staff-quality items; although this uncertainty is substantively informative, it also complicates scale construction and comparison. Finally, the multivariable models explained only a small share of variance, indicating that important determinants of institutional trust and reporting orientation were not measured in the present survey, such as prior exposure to child abuse, prior institutional contact, political trust, religiosity, stigma, family reputation concerns, rural service access, media literacy, or previous reporting experience.

## Conclusion

In conclusion, this study shows that among adult online respondents in Palestine and Jordan, endorsement of institutional involvement, multidisciplinary expertise, and perpetrator accountability was stronger than confidence in institutions and self-reported procedural knowledge. Cross-country differences were generally modest, whereas income was the most consistent measured correlate across outcomes. These findings should be interpreted cautiously because the sample was selective, online, and non-probability based, and because the models explained only a limited proportion of variance. Even so, the results suggest that strengthening child protection in Palestine and Jordan may require not only institutional capacity and legal frameworks, but also clearer public reporting pathways, stronger visibility of child-protection personnel and services, and continued work to translate symbolic support into practical reporting readiness.

## Data Availability

The datasets generated and analyzed during the current study are not publicly available because they address a sensitive child-protection topic and may present contextual disclosure or re-identification risk if released openly. De-identified data may be made available by the corresponding author on reasonable request, subject to ethical review and institutional approval.
