# Supplementary material for: Adult respondents’ perceptions of child abuse and formal child-protection institutions in Palestine and Jordan: a cross-sectional online survey
Source: Front Public Health. 2026 Jul 21;14:1859966. doi: 10.3389/fpubh.2026.1859966 (PMC13433272; doi:10.3389/fpubh.2026.1859966)
Supplement: Supplementary file 1 [file Table_1.DOCX]

Supplementary Table S1. Questionnaire domains, item sources, scoring, valid N, and internal consistency

| **Domain** | **Items included** | **No. of items** | **Original source / basis** | **Adaptation notes** | **Primary scoring** | **Composite valid N** | **Cronbach alpha (95% CI)** |
| --- | --- | --- | --- | --- | --- | --- | --- |
| Institutional trust and legitimacy | Confidence in institutions; Staff enjoy high social status; Would like to work in institution; Staff have high professionalism; Feels emotionally close to institutions | 5 | Mukai et al. cross-national public-perception dataset | Arabic wording adapted for Palestine/Jordan; translated into Arabic and back-translated into English by an expert; reviewed by one pediatric specialist and one Arabic/English language expert; piloted among 30 participants before main data collection. | 1–6 agreement/confidence; standardized to 0–100 | 779 | 0.743 (0.708–0.774) |
| Required staff expertise | Education and training; Psychology; Social welfare; Health and medicine; Law; Economics; Religion | 7 | Mukai et al. cross-national public-perception dataset | Arabic wording adapted for Palestine/Jordan; translated into Arabic and back-translated into English by an expert; reviewed by one pediatric specialist and one Arabic/English language expert; piloted among 30 participants before main data collection. | 1–6 importance; standardized to 0–100 | 779 | 0.919 (0.905–0.930) |
| Perceived staff qualities | Confident; Intelligent; Competent; Effective; Skilled; Friendly; Honest; Collaborative; Kind-hearted | 9 | Mukai et al. cross-national public-perception dataset | Arabic wording adapted for Palestine/Jordan; translated into Arabic and back-translated into English by an expert; reviewed by one pediatric specialist and one Arabic/English language expert; piloted among 30 participants before main data collection. | 1–6 applicability; 'I do not know/cannot say' excluded from primary score; ≥5/9 rated items required | 546 | 0.986 (0.982–0.989) |
| Perpetrator accountability | Physical abuse; Psychological abuse; Sexual abuse; Neglect; Exposure to domestic violence | 5 | Mukai et al.; wording culturally informed by ICAST-R literature | Arabic wording adapted for Palestine/Jordan; translated into Arabic and back-translated into English by an expert; reviewed by one pediatric specialist and one Arabic/English language expert; piloted among 30 participants before main data collection. | Perpetrator attribution=1; other attribution=0; multiplied by 100 | 779 | 0.825 (0.800–0.848) |
| Institutional response orientation | Staff have high experience/knowledge; Would contact institution if abuse suspected; Contacting institution is in child’s best interest; Contacting institution is in parents’ best interest; Sufficient knowledge of institutions; Understands how to respond to suspected abuse | 6 | LeCroy and Milligan-LeCroy; Dhooper et al.; Daro and Gelles | Arabic wording adapted for Palestine/Jordan; translated into Arabic and back-translated into English by an expert; reviewed by one pediatric specialist and one Arabic/English language expert; piloted among 30 participants before main data collection. | 1–6 agreement; standardized to 0–100 | 779 | 0.836 (0.813–0.856) |
| Media exposure | Social media exposure; Newspaper exposure; Television exposure; Online news exposure; Other media exposure | 5 | Mukai et al. cross-national public-perception dataset | Arabic wording adapted for Palestine/Jordan; translated into Arabic and back-translated into English by an expert; reviewed by one pediatric specialist and one Arabic/English language expert; piloted among 30 participants before main data collection. | 1–6 exposure; standardized to 0–100; ≥3/5 valid items required | 773 | 0.866 (0.849–0.883) |
| Punitive orientation | Sentences should be harsher; Would impose harsher penalties; Punishments are too lenient; Sentences are too short; Harsher punishment is better | 5 | Mukai and Fujino Attitudes toward Criminal Justice Scale, harsher-punishment dimension | Arabic wording adapted for Palestine/Jordan; translated into Arabic and back-translated into English by an expert; reviewed by one pediatric specialist and one Arabic/English language expert; piloted among 30 participants before main data collection. | 1–6 agreement; standardized to 0–100; ≥4/5 valid items required | 778 | 0.925 (0.912–0.937) |

Supplementary Table S2. Item-level missingness, uncertainty responses, and endorsement patterns

| **Domain** | **Item** | **Valid N** | **Missing N** | **Missing % of total** | **Uncertainty N** | **Uncertainty % of total** | **Mean 0–100** | **SD 0–100** | **Top-box/perpetrator N** | **Top-box/perpetrator % of valid** |
| --- | --- | --- | --- | --- | --- | --- | --- | --- | --- | --- |
| Institutional trust and legitimacy | Confidence in institutions | 779 | 0 | 0 | 0 | 0 | 45.88 | 27.11 | 126 | 16.2 |
| Institutional trust and legitimacy | Staff enjoy high social status | 779 | 0 | 0 | 0 | 0 | 48.65 | 20.5 | 87 | 11.2 |
| Institutional trust and legitimacy | Would like to work in institution | 779 | 0 | 0 | 0 | 0 | 48.42 | 26.23 | 143 | 18.4 |
| Institutional trust and legitimacy | Staff have high professionalism | 779 | 0 | 0 | 0 | 0 | 50.55 | 21.87 | 111 | 14.2 |
| Institutional trust and legitimacy | Feels emotionally close to institutions | 779 | 0 | 0 | 0 | 0 | 55.28 | 26.29 | 217 | 27.9 |
| Required staff expertise | Education and training | 779 | 0 | 0 | 0 | 0 | 78.36 | 22.77 | 504 | 64.7 |
| Required staff expertise | Psychology | 779 | 0 | 0 | 0 | 0 | 79.85 | 22.92 | 531 | 68.2 |
| Required staff expertise | Social welfare | 779 | 0 | 0 | 0 | 0 | 79.79 | 22.58 | 538 | 69.1 |
| Required staff expertise | Health and medicine | 779 | 0 | 0 | 0 | 0 | 70.99 | 23.24 | 399 | 51.2 |
| Required staff expertise | Law | 779 | 0 | 0 | 0 | 0 | 72.25 | 24.05 | 432 | 55.5 |
| Required staff expertise | Economics | 779 | 0 | 0 | 0 | 0 | 55.92 | 26.8 | 234 | 30 |
| Required staff expertise | Religion | 779 | 0 | 0 | 0 | 0 | 75.84 | 24.68 | 475 | 61 |
| Perceived staff qualities | Confident | 475 | 0 | 0 | 304 | 39 | 55.79 | 25.27 | 150 | 31.6 |
| Perceived staff qualities | Intelligent | 494 | 0 | 0 | 285 | 36.6 | 54.17 | 24 | 132 | 26.7 |
| Perceived staff qualities | Competent | 521 | 0 | 0 | 258 | 33.1 | 55.05 | 25.56 | 149 | 28.6 |
| Perceived staff qualities | Effective | 537 | 0 | 0 | 242 | 31.1 | 55.12 | 26.02 | 154 | 28.7 |
| Perceived staff qualities | Skilled | 501 | 0 | 0 | 278 | 35.7 | 54.81 | 25.43 | 147 | 29.3 |
| Perceived staff qualities | Friendly | 539 | 0 | 0 | 240 | 30.8 | 60.3 | 27.05 | 208 | 38.6 |
| Perceived staff qualities | Honest | 499 | 0 | 0 | 280 | 35.9 | 57.35 | 26.76 | 168 | 33.7 |
| Perceived staff qualities | Collaborative | 542 | 0 | 0 | 237 | 30.4 | 59.15 | 26.02 | 189 | 34.9 |
| Perceived staff qualities | Kind-hearted | 509 | 0 | 0 | 270 | 34.7 | 59.65 | 26.16 | 180 | 35.4 |
| Perpetrator accountability | Physical abuse | 779 | 0 | 0 | 0 | 0 | 70.86 | 45.47 | 552 | 70.9 |
| Perpetrator accountability | Psychological abuse | 779 | 0 | 0 | 0 | 0 | 70.73 | 45.53 | 551 | 70.7 |
| Perpetrator accountability | Sexual abuse | 779 | 0 | 0 | 0 | 0 | 66.75 | 47.14 | 520 | 66.8 |
| Perpetrator accountability | Neglect | 779 | 0 | 0 | 0 | 0 | 69.58 | 46.04 | 542 | 69.6 |
| Perpetrator accountability | Exposure to domestic violence | 779 | 0 | 0 | 0 | 0 | 61.49 | 48.69 | 479 | 61.5 |
| Institutional response orientation | Staff have high experience/knowledge | 779 | 0 | 0 | 0 | 0 | 54.97 | 21.61 | 168 | 21.6 |
| Institutional response orientation | Would contact institution if abuse suspected | 779 | 0 | 0 | 0 | 0 | 63.8 | 25.35 | 305 | 39.2 |
| Institutional response orientation | Contacting institution is in child’s best interest | 779 | 0 | 0 | 0 | 0 | 66.57 | 25.04 | 341 | 43.8 |
| Institutional response orientation | Contacting institution is in parents’ best interest | 779 | 0 | 0 | 0 | 0 | 59.67 | 24.91 | 247 | 31.7 |
| Institutional response orientation | Sufficient knowledge of institutions | 779 | 0 | 0 | 0 | 0 | 43.08 | 26.18 | 110 | 14.1 |
| Institutional response orientation | Understands how to respond to suspected abuse | 779 | 0 | 0 | 0 | 0 | 50.78 | 25.77 | 167 | 21.4 |
| Media exposure | Social media exposure | 776 | 3 | 0.4 | 0 | 0 | 47.58 | 30.43 | 189 | 24.4 |
| Media exposure | Newspaper exposure | 769 | 10 | 1.3 | 0 | 0 | 26.63 | 25.11 | 47 | 6.1 |
| Media exposure | Television exposure | 765 | 14 | 1.8 | 0 | 0 | 39.14 | 27.78 | 109 | 14.2 |
| Media exposure | Online news exposure | 774 | 5 | 0.6 | 0 | 0 | 47.39 | 30.27 | 184 | 23.8 |
| Media exposure | Other media exposure | 767 | 12 | 1.5 | 0 | 0 | 37.5 | 28.83 | 102 | 13.3 |
| Punitive orientation | Sentences should be harsher | 778 | 1 | 0.1 | 0 | 0 | 76.56 | 25.88 | 497 | 63.9 |
| Punitive orientation | Would impose harsher penalties | 778 | 1 | 0.1 | 0 | 0 | 75.4 | 25.45 | 472 | 60.7 |
| Punitive orientation | Punishments are too lenient | 775 | 4 | 0.5 | 0 | 0 | 72.57 | 26.48 | 456 | 58.8 |
| Punitive orientation | Sentences are too short | 777 | 2 | 0.3 | 0 | 0 | 66.1 | 29.4 | 373 | 48 |
| Punitive orientation | Harsher punishment is better | 777 | 2 | 0.3 | 0 | 0 | 76.09 | 25.67 | 492 | 63.3 |

Supplementary Table S3. Item-total correlations and alpha-if-item-deleted statistics

| **Domain** | **Item** | **Complete-case N for scale reliability** | **Item-total correlation** | **Corrected item-total correlation** | **Alpha if item deleted** | **Scale alpha (95% CI)** |
| --- | --- | --- | --- | --- | --- | --- |
| Institutional trust and legitimacy | Confidence in institutions | 779 | 0.655 | 0.41 | 0.739 | 0.743 (0.708–0.774) |
| Institutional trust and legitimacy | Staff enjoy high social status | 779 | 0.714 | 0.563 | 0.685 | 0.743 (0.708–0.774) |
| Institutional trust and legitimacy | Would like to work in institution | 779 | 0.634 | 0.392 | 0.743 | 0.743 (0.708–0.774) |
| Institutional trust and legitimacy | Staff have high professionalism | 779 | 0.77 | 0.629 | 0.658 | 0.743 (0.708–0.774) |
| Institutional trust and legitimacy | Feels emotionally close to institutions | 779 | 0.775 | 0.596 | 0.662 | 0.743 (0.708–0.774) |
| Required staff expertise | Education and training | 779 | 0.856 | 0.8 | 0.902 | 0.919 (0.905–0.930) |
| Required staff expertise | Psychology | 779 | 0.875 | 0.826 | 0.899 | 0.919 (0.905–0.930) |
| Required staff expertise | Social welfare | 779 | 0.884 | 0.839 | 0.898 | 0.919 (0.905–0.930) |
| Required staff expertise | Health and medicine | 779 | 0.843 | 0.781 | 0.904 | 0.919 (0.905–0.930) |
| Required staff expertise | Law | 779 | 0.85 | 0.788 | 0.903 | 0.919 (0.905–0.930) |
| Required staff expertise | Economics | 779 | 0.652 | 0.517 | 0.933 | 0.919 (0.905–0.930) |
| Required staff expertise | Religion | 779 | 0.822 | 0.748 | 0.907 | 0.919 (0.905–0.930) |
| Perceived staff qualities | Confident | 297 | 0.92 | 0.899 | 0.986 | 0.986 (0.982–0.989) |
| Perceived staff qualities | Intelligent | 297 | 0.929 | 0.912 | 0.985 | 0.986 (0.982–0.989) |
| Perceived staff qualities | Competent | 297 | 0.941 | 0.924 | 0.985 | 0.986 (0.982–0.989) |
| Perceived staff qualities | Effective | 297 | 0.957 | 0.945 | 0.984 | 0.986 (0.982–0.989) |
| Perceived staff qualities | Skilled | 297 | 0.956 | 0.944 | 0.984 | 0.986 (0.982–0.989) |
| Perceived staff qualities | Friendly | 297 | 0.961 | 0.949 | 0.984 | 0.986 (0.982–0.989) |
| Perceived staff qualities | Honest | 297 | 0.959 | 0.947 | 0.984 | 0.986 (0.982–0.989) |
| Perceived staff qualities | Collaborative | 297 | 0.965 | 0.954 | 0.984 | 0.986 (0.982–0.989) |
| Perceived staff qualities | Kind-hearted | 297 | 0.954 | 0.941 | 0.984 | 0.986 (0.982–0.989) |
| Perpetrator accountability | Physical abuse | 779 | 0.787 | 0.653 | 0.782 | 0.825 (0.800–0.848) |
| Perpetrator accountability | Psychological abuse | 779 | 0.785 | 0.65 | 0.783 | 0.825 (0.800–0.848) |
| Perpetrator accountability | Sexual abuse | 779 | 0.783 | 0.64 | 0.785 | 0.825 (0.800–0.848) |
| Perpetrator accountability | Neglect | 779 | 0.794 | 0.662 | 0.779 | 0.825 (0.800–0.848) |
| Perpetrator accountability | Exposure to domestic violence | 779 | 0.694 | 0.506 | 0.825 | 0.825 (0.800–0.848) |
| Institutional response orientation | Staff have high experience/knowledge | 779 | 0.669 | 0.537 | 0.823 | 0.836 (0.813–0.856) |
| Institutional response orientation | Would contact institution if abuse suspected | 779 | 0.788 | 0.672 | 0.797 | 0.836 (0.813–0.856) |
| Institutional response orientation | Contacting institution is in child’s best interest | 779 | 0.797 | 0.686 | 0.794 | 0.836 (0.813–0.856) |
| Institutional response orientation | Contacting institution is in parents’ best interest | 779 | 0.754 | 0.627 | 0.806 | 0.836 (0.813–0.856) |
| Institutional response orientation | Sufficient knowledge of institutions | 779 | 0.674 | 0.509 | 0.831 | 0.836 (0.813–0.856) |
| Institutional response orientation | Understands how to respond to suspected abuse | 779 | 0.767 | 0.639 | 0.803 | 0.836 (0.813–0.856) |
| Media exposure | Social media exposure | 752 | 0.81 | 0.683 | 0.84 | 0.866 (0.849–0.883) |
| Media exposure | Newspaper exposure | 752 | 0.721 | 0.588 | 0.861 | 0.866 (0.849–0.883) |
| Media exposure | Television exposure | 752 | 0.849 | 0.755 | 0.822 | 0.866 (0.849–0.883) |
| Media exposure | Online news exposure | 752 | 0.868 | 0.772 | 0.816 | 0.866 (0.849–0.883) |
| Media exposure | Other media exposure | 752 | 0.783 | 0.651 | 0.847 | 0.866 (0.849–0.883) |
| Punitive orientation | Sentences should be harsher | 770 | 0.909 | 0.855 | 0.899 | 0.925 (0.912–0.937) |
| Punitive orientation | Would impose harsher penalties | 770 | 0.921 | 0.874 | 0.895 | 0.925 (0.912–0.937) |
| Punitive orientation | Punishments are too lenient | 770 | 0.895 | 0.831 | 0.903 | 0.925 (0.912–0.937) |
| Punitive orientation | Sentences are too short | 770 | 0.777 | 0.64 | 0.944 | 0.925 (0.912–0.937) |
| Punitive orientation | Harsher punishment is better | 770 | 0.907 | 0.853 | 0.899 | 0.925 (0.912–0.937) |

Supplementary Table S4. Exploratory factor-analysis and dimensionality diagnostics

| **Domain** | **Complete-case N** | **Items** | **KMO** | **Bartlett χ²** | **Bartlett df** | **Bartlett p** | **Eigenvalue 1** | **Eigenvalue 2** | **% variance factor 1** | **Interpretation** |
| --- | --- | --- | --- | --- | --- | --- | --- | --- | --- | --- |
| Institutional trust and legitimacy | 779 | 5 | 0.761 | 942.27 | 10 | <0.001 | 2.556 | 0.883 | 51.12 | Dominant one-factor pattern |
| Required staff expertise | 779 | 7 | 0.91 | 4341.09 | 21 | <0.001 | 4.833 | 0.84 | 69.04 | Dominant one-factor pattern |
| Perceived staff qualities | 297 | 9 | 0.951 | 4943.63 | 36 | <0.001 | 8.106 | 0.261 | 90.06 | Dominant one-factor pattern |
| Perpetrator accountability | 779 | 5 | 0.84 | 1326.67 | 10 | <0.001 | 2.964 | 0.709 | 59.28 | Dominant one-factor pattern |
| Institutional response orientation | 779 | 6 | 0.782 | 2006.53 | 15 | <0.001 | 3.319 | 1.033 | 55.31 | Possible multidimensionality; inspect factor structure |
| Media exposure | 752 | 5 | 0.826 | 1820.71 | 10 | <0.001 | 3.266 | 0.658 | 65.32 | Dominant one-factor pattern |
| Punitive orientation | 770 | 5 | 0.888 | 3379.79 | 10 | <0.001 | 3.908 | 0.533 | 78.17 | Dominant one-factor pattern |
| Table S4b. Rotated two-factor solution for institutional response orientation |  |  |  |  |  |  |  |  |  |  |
| Institutional response orientation item | Rotated factor 1 loading | Rotated factor 2 loading | Suggested interpretation |  |  |  |  |  |  |  |
| Staff have high experience/knowledge | 0.655 | 0.246 | Normative/pro-intervention |  |  |  |  |  |  |  |
| Would contact institution if abuse suspected | 0.834 | 0.207 | Normative/pro-intervention |  |  |  |  |  |  |  |
| Contacting institution is in child’s best interest | 0.892 | 0.145 | Normative/pro-intervention |  |  |  |  |  |  |  |
| Contacting institution is in parents’ best interest | 0.733 | 0.277 | Normative/pro-intervention |  |  |  |  |  |  |  |
| Sufficient knowledge of institutions | 0.136 | 0.926 | Procedural knowledge/competence |  |  |  |  |  |  |  |
| Understands how to respond to suspected abuse | 0.327 | 0.843 | Procedural knowledge/competence |  |  |  |  |  |  |  |

Supplementary Table S5. Comparison of respondents included versus excluded from the perceived staff qualities score

| **Variable** | **Category** | **Included in staff-quality score n (%)** | **Excluded from staff-quality score n (%)** | **p-value** |
| --- | --- | --- | --- | --- |
| Age, years | Mean ± SD | 29.40 ± 11.49 | 30.05 ± 11.22 | 0.468 |
| Country | Palestine | 269 (49.3) | 122 (52.4) | 0.476 |
|  | Jordan | 277 (50.7) | 111 (47.6) |  |
| Age group | 18–35 years | 414 (75.8) | 169 (72.5) | 0.051 |
|  | 36–50 years | 84 (15.4) | 51 (21.9) |  |
|  | Over 50 years | 46 (8.4) | 13 (5.6) |  |
| Sex | Male | 236 (43.2) | 98 (42.1) | 0.825 |
|  | Female | 310 (56.8) | 135 (57.9) |  |
| Education | Secondary or lower | 43 (7.9) | 14 (6.0) | 0.444 |
|  | University and above | 503 (92.1) | 219 (94.0) |  |
| Income | Low | 95 (17.4) | 43 (18.5) | 0.939 |
|  | Average | 327 (59.9) | 138 (59.2) |  |
|  | High | 124 (22.7) | 52 (22.3) |  |
| Employment | Student | 252 (46.2) | 94 (40.3) | 0.652 |
|  | Government employee | 133 (24.4) | 59 (25.3) |  |
|  | Private sector | 61 (11.2) | 34 (14.6) |  |
|  | Part-time work | 29 (5.3) | 10 (4.3) |  |
|  | Not working | 30 (5.5) | 17 (7.3) |  |
|  | Housewife | 22 (4.0) | 10 (4.3) |  |
|  | Retired | 19 (3.5) | 9 (3.9) |  |
| Residence | City | 416 (76.2) | 171 (73.4) | 0.529 |
|  | Village | 116 (21.2) | 53 (22.7) |  |
|  | Camp/Countryside | 14 (2.6) | 9 (3.9) |  |
| Marital status | Single | 352 (64.5) | 147 (63.1) | 0.585 |
|  | Married | 184 (33.7) | 78 (33.5) |  |
|  | Divorced | 8 (1.5) | 6 (2.6) |  |
|  | Widowed | 2 (0.4) | 2 (0.9) |  |
| Parenthood | No | 392 (71.8) | 158 (67.8) | 0.302 |
|  | Yes | 154 (28.2) | 75 (32.2) |  |

Supplementary Table S6. Sensitivity analyses for perceived staff qualities scoring

| **Strategy** | **Scoring rule** | **Scored N** | **Overall mean ± SD** | **Palestine mean ± SD** | **Jordan mean ± SD** | **Adjusted country effect B (95% CI)** | **p-value** | **Interpretation** |
| --- | --- | --- | --- | --- | --- | --- | --- | --- |
| Primary rule reproducing manuscript | Uncertainty treated as missing; score calculated if ≥5/9 rated items | 546 | 56.61 ± 23.82 | 56.89 ± 22.74 (n=269) | 56.34 ± 24.85 (n=277) | 0.02 (-4.00 to 4.04) | 0.993 | No meaningful Palestine–Jordan difference |
| Stricter threshold | Uncertainty treated as missing; score calculated if ≥7/9 rated items | 430 | 57.80 ± 25.41 | 57.79 ± 24.50 (n=203) | 57.81 ± 26.26 (n=227) | 0.29 (-4.57 to 5.15) | 0.906 | No meaningful Palestine–Jordan difference |
| Very strict threshold | Uncertainty treated as missing; score calculated if ≥8/9 rated items | 369 | 58.96 ± 26.20 | 58.65 ± 25.33 (n=170) | 59.22 ± 26.98 (n=199) | 0.90 (-4.51 to 6.31) | 0.744 | No meaningful Palestine–Jordan difference |
| Uncertainty coded to midpoint | Uncertainty responses coded as 3.5/6; all 9 items used | 779 | 54.52 ± 19.10 | 54.40 ± 17.78 (n=391) | 54.64 ± 20.37 (n=388) | 0.57 (-2.12 to 3.26) | 0.676 | No meaningful Palestine–Jordan difference |

Supplementary Table S7. Full multivariable linear regression models and diagnostics

| **Outcome** | **Predictor** | **B** | **95% CI lower** | **95% CI upper** | **Standardized β** | **p-value** | **VIF** | **Model N** | **R²** | **Adjusted R²** | **Breusch–Pagan p** |
| --- | --- | --- | --- | --- | --- | --- | --- | --- | --- | --- | --- |
| Institutional trust and legitimacy | Intercept | 42.5 | 36.59 | 48.42 |  | <0.001 |  | 777 | 0.034 | 0.025 | 0.345 |
| Institutional trust and legitimacy | Country: Jordan vs Palestine | 2.06 | -0.38 | 4.49 | 0.06 | 0.098 | 1.03 | 777 | 0.034 | 0.025 | 0.345 |
| Institutional trust and legitimacy | Sex: Female vs Male | 1.98 | -0.48 | 4.43 | 0.057 | 0.115 | 1.03 | 777 | 0.034 | 0.025 | 0.345 |
| Institutional trust and legitimacy | Education: University+ vs Secondary/lower | 1.63 | -3.1 | 6.36 | 0.024 | 0.500 | 1.03 | 777 | 0.034 | 0.025 | 0.345 |
| Institutional trust and legitimacy | Income: Average vs Low | 6.51 | 3.23 | 9.8 | 0.186 | <0.001 | 1.81 | 777 | 0.034 | 0.025 | 0.345 |
| Institutional trust and legitimacy | Income: High vs Low | 2.1 | -1.76 | 5.95 | 0.051 | 0.286 | 1.81 | 777 | 0.034 | 0.025 | 0.345 |
| Institutional trust and legitimacy | Age (years) | -0.03 | -0.16 | 0.11 | -0.017 | 0.724 | 1.76 | 777 | 0.034 | 0.025 | 0.345 |
| Institutional trust and legitimacy | Has children: Yes vs No | -0.35 | -3.81 | 3.11 | -0.009 | 0.843 | 1.74 | 777 | 0.034 | 0.025 | 0.345 |
| Required staff expertise | Intercept | 72.16 | 65.4 | 78.93 |  | <0.001 |  | 777 | 0.028 | 0.019 | 0.046 |
| Required staff expertise | Country: Jordan vs Palestine | 0.14 | -2.65 | 2.92 | 0.004 | 0.922 | 1.03 | 777 | 0.028 | 0.019 | 0.046 |
| Required staff expertise | Sex: Female vs Male | 3.26 | 0.45 | 6.08 | 0.082 | 0.023 | 1.03 | 777 | 0.028 | 0.019 | 0.046 |
| Required staff expertise | Education: University+ vs Secondary/lower | -1.27 | -6.68 | 4.14 | -0.017 | 0.645 | 1.03 | 777 | 0.028 | 0.019 | 0.046 |
| Required staff expertise | Income: Average vs Low | 5.67 | 1.92 | 9.43 | 0.142 | 0.003 | 1.81 | 777 | 0.028 | 0.019 | 0.046 |
| Required staff expertise | Income: High vs Low | 6.81 | 2.4 | 11.22 | 0.145 | 0.003 | 1.81 | 777 | 0.028 | 0.019 | 0.046 |
| Required staff expertise | Age (years) | -0.19 | -0.35 | -0.03 | -0.11 | 0.020 | 1.76 | 777 | 0.028 | 0.019 | 0.046 |
| Required staff expertise | Has children: Yes vs No | 3.55 | -0.41 | 7.51 | 0.083 | 0.079 | 1.74 | 777 | 0.028 | 0.019 | 0.046 |
| Perceived staff qualities | Intercept | 55.29 | 45.75 | 64.83 |  | <0.001 |  | 544 | 0.054 | 0.041 | 0.197 |
| Perceived staff qualities | Country: Jordan vs Palestine | 0.02 | -4 | 4.04 | 0 | 0.993 | 1.05 | 544 | 0.054 | 0.041 | 0.197 |
| Perceived staff qualities | Sex: Female vs Male | 0.68 | -3.4 | 4.75 | 0.014 | 0.744 | 1.06 | 544 | 0.054 | 0.041 | 0.197 |
| Perceived staff qualities | Education: University+ vs Secondary/lower | 5.41 | -2.19 | 13.01 | 0.06 | 0.163 | 1.05 | 544 | 0.054 | 0.041 | 0.197 |
| Perceived staff qualities | Income: Average vs Low | 9.86 | 4.46 | 15.27 | 0.203 | <0.001 | 1.82 | 544 | 0.054 | 0.041 | 0.197 |
| Perceived staff qualities | Income: High vs Low | 4.47 | -1.9 | 10.84 | 0.079 | 0.168 | 1.85 | 544 | 0.054 | 0.041 | 0.197 |
| Perceived staff qualities | Age (years) | -0.42 | -0.64 | -0.2 | -0.204 | <0.001 | 1.65 | 544 | 0.054 | 0.041 | 0.197 |
| Perceived staff qualities | Has children: Yes vs No | 4.56 | -1.02 | 10.15 | 0.087 | 0.109 | 1.65 | 544 | 0.054 | 0.041 | 0.197 |
| Perpetrator accountability | Intercept | 53.65 | 41.28 | 66.03 |  | <0.001 |  | 777 | 0.016 | 0.007 | 0.610 |
| Perpetrator accountability | Country: Jordan vs Palestine | -3.66 | -8.75 | 1.44 | -0.051 | 0.159 | 1.03 | 777 | 0.016 | 0.007 | 0.610 |
| Perpetrator accountability | Sex: Female vs Male | -1.01 | -6.15 | 4.13 | -0.014 | 0.700 | 1.03 | 777 | 0.016 | 0.007 | 0.610 |
| Perpetrator accountability | Education: University+ vs Secondary/lower | 13.83 | 3.94 | 23.73 | 0.099 | 0.006 | 1.03 | 777 | 0.016 | 0.007 | 0.610 |
| Perpetrator accountability | Income: Average vs Low | 1.24 | -5.63 | 8.11 | 0.017 | 0.724 | 1.81 | 777 | 0.016 | 0.007 | 0.610 |
| Perpetrator accountability | Income: High vs Low | 2.59 | -5.47 | 10.66 | 0.03 | 0.528 | 1.81 | 777 | 0.016 | 0.007 | 0.610 |
| Perpetrator accountability | Age (years) | 0.05 | -0.25 | 0.34 | 0.014 | 0.760 | 1.76 | 777 | 0.016 | 0.007 | 0.610 |
| Perpetrator accountability | Has children: Yes vs No | 3.9 | -3.34 | 11.14 | 0.05 | 0.290 | 1.74 | 777 | 0.016 | 0.007 | 0.610 |
| Institutional response orientation | Intercept | 53.91 | 47.56 | 60.26 |  | <0.001 |  | 777 | 0.022 | 0.013 | 0.548 |
| Institutional response orientation | Country: Jordan vs Palestine | 0.97 | -1.64 | 3.59 | 0.027 | 0.465 | 1.03 | 777 | 0.022 | 0.013 | 0.548 |
| Institutional response orientation | Sex: Female vs Male | 2.61 | -0.03 | 5.25 | 0.07 | 0.052 | 1.03 | 777 | 0.022 | 0.013 | 0.548 |
| Institutional response orientation | Education: University+ vs Secondary/lower | -3.11 | -8.19 | 1.97 | -0.043 | 0.230 | 1.03 | 777 | 0.022 | 0.013 | 0.548 |
| Institutional response orientation | Income: Average vs Low | 5.53 | 2 | 9.06 | 0.148 | 0.002 | 1.81 | 777 | 0.022 | 0.013 | 0.548 |
| Institutional response orientation | Income: High vs Low | 3.53 | -0.61 | 7.67 | 0.08 | 0.094 | 1.81 | 777 | 0.022 | 0.013 | 0.548 |
| Institutional response orientation | Age (years) | -0.04 | -0.19 | 0.11 | -0.023 | 0.627 | 1.76 | 777 | 0.022 | 0.013 | 0.548 |
| Institutional response orientation | Has children: Yes vs No | 1.39 | -2.33 | 5.11 | 0.034 | 0.463 | 1.74 | 777 | 0.022 | 0.013 | 0.548 |
| Media exposure | Intercept | 38.06 | 30.09 | 46.02 |  | <0.001 |  | 771 | 0.018 | 0.009 | 0.003 |
| Media exposure | Country: Jordan vs Palestine | 0.24 | -3.05 | 3.53 | 0.005 | 0.886 | 1.03 | 771 | 0.018 | 0.009 | 0.003 |
| Media exposure | Sex: Female vs Male | 3.21 | -0.11 | 6.53 | 0.069 | 0.058 | 1.03 | 771 | 0.018 | 0.009 | 0.003 |
| Media exposure | Education: University+ vs Secondary/lower | -1.24 | -7.61 | 5.12 | -0.014 | 0.702 | 1.03 | 771 | 0.018 | 0.009 | 0.003 |
| Media exposure | Income: Average vs Low | 3.01 | -1.43 | 7.44 | 0.064 | 0.184 | 1.81 | 771 | 0.018 | 0.009 | 0.003 |
| Media exposure | Income: High vs Low | -3.46 | -8.66 | 1.75 | -0.063 | 0.193 | 1.81 | 771 | 0.018 | 0.009 | 0.003 |
| Media exposure | Age (years) | 0 | -0.19 | 0.19 | 0 | 0.997 | 1.77 | 771 | 0.018 | 0.009 | 0.003 |
| Media exposure | Has children: Yes vs No | -1.28 | -5.98 | 3.43 | -0.025 | 0.594 | 1.75 | 771 | 0.018 | 0.009 | 0.003 |
| Punitive orientation | Intercept | 68.07 | 60.03 | 76.1 |  | <0.001 |  | 776 | 0.031 | 0.022 | 0.093 |
| Punitive orientation | Country: Jordan vs Palestine | -1.32 | -4.64 | 1.99 | -0.028 | 0.433 | 1.03 | 776 | 0.031 | 0.022 | 0.093 |
| Punitive orientation | Sex: Female vs Male | 2.56 | -0.79 | 5.9 | 0.054 | 0.134 | 1.03 | 776 | 0.031 | 0.022 | 0.093 |
| Punitive orientation | Education: University+ vs Secondary/lower | -0.87 | -7.29 | 5.56 | -0.01 | 0.791 | 1.03 | 776 | 0.031 | 0.022 | 0.093 |
| Punitive orientation | Income: Average vs Low | 8.38 | 3.92 | 12.85 | 0.176 | <0.001 | 1.81 | 776 | 0.031 | 0.022 | 0.093 |
| Punitive orientation | Income: High vs Low | 9.79 | 4.55 | 15.03 | 0.175 | <0.001 | 1.81 | 776 | 0.031 | 0.022 | 0.093 |
| Punitive orientation | Age (years) | -0.11 | -0.3 | 0.08 | -0.056 | 0.238 | 1.76 | 776 | 0.031 | 0.022 | 0.093 |
| Punitive orientation | Has children: Yes vs No | 4.81 | 0.1 | 9.52 | 0.094 | 0.045 | 1.74 | 776 | 0.031 | 0.022 | 0.093 |

Supplementary Table S8. Country-by-sociodemographic interaction tests

| **Outcome** | **Interaction term added to main model** | **Interaction B** | **p-value** | **Model N** | **Multiplicity-aware interpretation** |
| --- | --- | --- | --- | --- | --- |
| Institutional trust and legitimacy | Country (Jordan) × Sex | 0.852 | 0.734 | 777 | Exploratory; none should be emphasized unless corrected/multiplicity-aware significance is retained |
| Institutional trust and legitimacy | Country (Jordan) × Age | 0.074 | 0.509 | 777 | Exploratory; none should be emphasized unless corrected/multiplicity-aware significance is retained |
| Institutional trust and legitimacy | Country (Jordan) × University education | -2.88 | 0.553 | 777 | Exploratory; none should be emphasized unless corrected/multiplicity-aware significance is retained |
| Institutional trust and legitimacy | Country (Jordan) × Average income | -2.128 | 0.394 | 777 | Exploratory; none should be emphasized unless corrected/multiplicity-aware significance is retained |
| Institutional trust and legitimacy | Country (Jordan) × High income | 2.666 | 0.365 | 777 | Exploratory; none should be emphasized unless corrected/multiplicity-aware significance is retained |
| Institutional trust and legitimacy | Country (Jordan) × Parenthood | 0.557 | 0.838 | 777 | Exploratory; none should be emphasized unless corrected/multiplicity-aware significance is retained |
| Required staff expertise | Country (Jordan) × Sex | 5.58 | 0.052 | 777 | Exploratory; none should be emphasized unless corrected/multiplicity-aware significance is retained |
| Required staff expertise | Country (Jordan) × Age | 0.101 | 0.430 | 777 | Exploratory; none should be emphasized unless corrected/multiplicity-aware significance is retained |
| Required staff expertise | Country (Jordan) × University education | 3.753 | 0.499 | 777 | Exploratory; none should be emphasized unless corrected/multiplicity-aware significance is retained |
| Required staff expertise | Country (Jordan) × Average income | -1.097 | 0.701 | 777 | Exploratory; none should be emphasized unless corrected/multiplicity-aware significance is retained |
| Required staff expertise | Country (Jordan) × High income | 1.557 | 0.644 | 777 | Exploratory; none should be emphasized unless corrected/multiplicity-aware significance is retained |
| Required staff expertise | Country (Jordan) × Parenthood | 2.239 | 0.473 | 777 | Exploratory; none should be emphasized unless corrected/multiplicity-aware significance is retained |
| Perceived staff qualities | Country (Jordan) × Sex | 2.038 | 0.621 | 544 | Exploratory; none should be emphasized unless corrected/multiplicity-aware significance is retained |
| Perceived staff qualities | Country (Jordan) × Age | -0.06 | 0.745 | 544 | Exploratory; none should be emphasized unless corrected/multiplicity-aware significance is retained |
| Perceived staff qualities | Country (Jordan) × University education | 3.379 | 0.659 | 544 | Exploratory; none should be emphasized unless corrected/multiplicity-aware significance is retained |
| Perceived staff qualities | Country (Jordan) × Average income | -4.141 | 0.311 | 544 | Exploratory; none should be emphasized unless corrected/multiplicity-aware significance is retained |
| Perceived staff qualities | Country (Jordan) × High income | 4.855 | 0.313 | 544 | Exploratory; none should be emphasized unless corrected/multiplicity-aware significance is retained |
| Perceived staff qualities | Country (Jordan) × Parenthood | -1.756 | 0.698 | 544 | Exploratory; none should be emphasized unless corrected/multiplicity-aware significance is retained |
| Perpetrator accountability | Country (Jordan) × Sex | 0.161 | 0.976 | 777 | Exploratory; none should be emphasized unless corrected/multiplicity-aware significance is retained |
| Perpetrator accountability | Country (Jordan) × Age | 0.117 | 0.616 | 777 | Exploratory; none should be emphasized unless corrected/multiplicity-aware significance is retained |
| Perpetrator accountability | Country (Jordan) × University education | -4.275 | 0.674 | 777 | Exploratory; none should be emphasized unless corrected/multiplicity-aware significance is retained |
| Perpetrator accountability | Country (Jordan) × Average income | 3.653 | 0.484 | 777 | Exploratory; none should be emphasized unless corrected/multiplicity-aware significance is retained |
| Perpetrator accountability | Country (Jordan) × High income | -0.017 | 0.998 | 777 | Exploratory; none should be emphasized unless corrected/multiplicity-aware significance is retained |
| Perpetrator accountability | Country (Jordan) × Parenthood | 2.464 | 0.666 | 777 | Exploratory; none should be emphasized unless corrected/multiplicity-aware significance is retained |
| Institutional response orientation | Country (Jordan) × Sex | 0.091 | 0.973 | 777 | Exploratory; none should be emphasized unless corrected/multiplicity-aware significance is retained |
| Institutional response orientation | Country (Jordan) × Age | -0.112 | 0.351 | 777 | Exploratory; none should be emphasized unless corrected/multiplicity-aware significance is retained |
| Institutional response orientation | Country (Jordan) × University education | 4.058 | 0.436 | 777 | Exploratory; none should be emphasized unless corrected/multiplicity-aware significance is retained |
| Institutional response orientation | Country (Jordan) × Average income | -3.643 | 0.174 | 777 | Exploratory; none should be emphasized unless corrected/multiplicity-aware significance is retained |
| Institutional response orientation | Country (Jordan) × High income | 1.894 | 0.549 | 777 | Exploratory; none should be emphasized unless corrected/multiplicity-aware significance is retained |
| Institutional response orientation | Country (Jordan) × Parenthood | -5.797 | 0.048 | 777 | Exploratory; none should be emphasized unless corrected/multiplicity-aware significance is retained |
| Media exposure | Country (Jordan) × Sex | -2.568 | 0.449 | 771 | Exploratory; none should be emphasized unless corrected/multiplicity-aware significance is retained |
| Media exposure | Country (Jordan) × Age | 0.013 | 0.933 | 771 | Exploratory; none should be emphasized unless corrected/multiplicity-aware significance is retained |
| Media exposure | Country (Jordan) × University education | 9.972 | 0.126 | 771 | Exploratory; none should be emphasized unless corrected/multiplicity-aware significance is retained |
| Media exposure | Country (Jordan) × Average income | 0.737 | 0.827 | 771 | Exploratory; none should be emphasized unless corrected/multiplicity-aware significance is retained |
| Media exposure | Country (Jordan) × High income | 0.806 | 0.839 | 771 | Exploratory; none should be emphasized unless corrected/multiplicity-aware significance is retained |
| Media exposure | Country (Jordan) × Parenthood | -0.74 | 0.841 | 771 | Exploratory; none should be emphasized unless corrected/multiplicity-aware significance is retained |
| Punitive orientation | Country (Jordan) × Sex | 2.527 | 0.459 | 776 | Exploratory; none should be emphasized unless corrected/multiplicity-aware significance is retained |
| Punitive orientation | Country (Jordan) × Age | 0.184 | 0.226 | 776 | Exploratory; none should be emphasized unless corrected/multiplicity-aware significance is retained |
| Punitive orientation | Country (Jordan) × University education | 8.568 | 0.193 | 776 | Exploratory; none should be emphasized unless corrected/multiplicity-aware significance is retained |
| Punitive orientation | Country (Jordan) × Average income | 0.882 | 0.795 | 776 | Exploratory; none should be emphasized unless corrected/multiplicity-aware significance is retained |
| Punitive orientation | Country (Jordan) × High income | -0.47 | 0.907 | 776 | Exploratory; none should be emphasized unless corrected/multiplicity-aware significance is retained |
| Punitive orientation | Country (Jordan) × Parenthood | 1.422 | 0.702 | 776 | Exploratory; none should be emphasized unless corrected/multiplicity-aware significance is retained |

Supplementary Table S9. Full item-level cross-country comparisons

| **Domain** | **Item** | **Overall mean 0–100 ± SD** | **Palestine mean ± SD** | **Jordan mean ± SD** | **Mean difference P–J** | **p-value** |
| --- | --- | --- | --- | --- | --- | --- |
| Institutional trust and legitimacy | Confidence in institutions | 45.88 ± 27.11 | 41.89 ± 25.33 | 49.90 ± 28.27 | -8 | <0.001 |
| Institutional trust and legitimacy | Staff enjoy high social status | 48.65 ± 20.50 | 48.24 ± 20.32 | 49.07 ± 20.69 | -0.84 | 0.569 |
| Institutional trust and legitimacy | Would like to work in institution | 48.42 ± 26.23 | 47.26 ± 26.14 | 49.59 ± 26.29 | -2.32 | 0.216 |
| Institutional trust and legitimacy | Staff have high professionalism | 50.55 ± 21.87 | 50.38 ± 21.62 | 50.72 ± 22.15 | -0.34 | 0.829 |
| Institutional trust and legitimacy | Feels emotionally close to institutions | 55.28 ± 26.29 | 54.94 ± 26.72 | 55.62 ± 25.89 | -0.68 | 0.717 |
| Required staff expertise | Education and training | 78.36 ± 22.77 | 79.08 ± 21.13 | 77.63 ± 24.33 | 1.45 | 0.375 |
| Required staff expertise | Psychology | 79.85 ± 22.92 | 80.61 ± 21.43 | 79.07 ± 24.34 | 1.54 | 0.349 |
| Required staff expertise | Social welfare | 79.79 ± 22.58 | 79.90 ± 21.00 | 79.69 ± 24.10 | 0.21 | 0.898 |
| Required staff expertise | Health and medicine | 70.99 ± 23.24 | 70.43 ± 22.64 | 71.55 ± 23.86 | -1.11 | 0.505 |
| Required staff expertise | Law | 72.25 ± 24.05 | 72.23 ± 22.98 | 72.27 ± 25.11 | -0.04 | 0.980 |
| Required staff expertise | Economics | 55.92 ± 26.80 | 54.37 ± 26.35 | 57.47 ± 27.18 | -3.1 | 0.106 |
| Required staff expertise | Religion | 75.84 ± 24.68 | 75.55 ± 23.39 | 76.13 ± 25.93 | -0.58 | 0.741 |
| Perceived staff qualities | Confident | 55.79 ± 25.27 | 55.35 ± 24.43 | 56.19 ± 26.07 | -0.84 | 0.716 |
| Perceived staff qualities | Intelligent | 54.17 ± 24.00 | 55.23 ± 23.27 | 53.20 ± 24.66 | 2.03 | 0.347 |
| Perceived staff qualities | Competent | 55.05 ± 25.56 | 54.65 ± 25.33 | 55.43 ± 25.81 | -0.79 | 0.726 |
| Perceived staff qualities | Effective | 55.12 ± 26.02 | 54.07 ± 25.12 | 56.18 ± 26.90 | -2.11 | 0.349 |
| Perceived staff qualities | Skilled | 54.81 ± 25.43 | 54.83 ± 24.80 | 54.79 ± 26.03 | 0.04 | 0.987 |
| Perceived staff qualities | Friendly | 60.30 ± 27.05 | 61.13 ± 25.54 | 59.49 ± 28.46 | 1.64 | 0.481 |
| Perceived staff qualities | Honest | 57.35 ± 26.76 | 57.00 ± 26.28 | 57.68 ± 27.24 | -0.68 | 0.776 |
| Perceived staff qualities | Collaborative | 59.15 ± 26.02 | 59.20 ± 25.07 | 59.10 ± 27.02 | 0.1 | 0.965 |
| Perceived staff qualities | Kind-hearted | 59.65 ± 26.16 | 59.92 ± 25.06 | 59.39 ± 27.18 | 0.52 | 0.821 |
| Perpetrator accountability | Physical abuse | 70.86 ± 45.47 | 72.12 ± 44.90 | 69.59 ± 46.06 | 2.54 | 0.437 |
| Perpetrator accountability | Psychological abuse | 70.73 ± 45.53 | 72.12 ± 44.90 | 69.33 ± 46.17 | 2.79 | 0.392 |
| Perpetrator accountability | Sexual abuse | 66.75 ± 47.14 | 68.03 ± 46.70 | 65.46 ± 47.61 | 2.57 | 0.448 |
| Perpetrator accountability | Neglect | 69.58 ± 46.04 | 72.63 ± 44.64 | 66.49 ± 47.26 | 6.14 | 0.063 |
| Perpetrator accountability | Exposure to domestic violence | 61.49 ± 48.69 | 62.92 ± 48.36 | 60.05 ± 49.04 | 2.86 | 0.412 |
| Institutional response orientation | Staff have high experience/knowledge | 54.97 ± 21.61 | 54.07 ± 21.57 | 55.88 ± 21.65 | -1.81 | 0.243 |
| Institutional response orientation | Would contact institution if abuse suspected | 63.80 ± 25.35 | 63.79 ± 25.58 | 63.81 ± 25.15 | -0.03 | 0.987 |
| Institutional response orientation | Contacting institution is in child’s best interest | 66.57 ± 25.04 | 67.21 ± 24.69 | 65.93 ± 25.41 | 1.28 | 0.475 |
| Institutional response orientation | Contacting institution is in parents’ best interest | 59.67 ± 24.91 | 59.13 ± 25.02 | 60.21 ± 24.82 | -1.08 | 0.547 |
| Institutional response orientation | Sufficient knowledge of institutions | 43.08 ± 26.18 | 41.07 ± 26.21 | 45.10 ± 26.03 | -4.03 | 0.032 |
| Institutional response orientation | Understands how to respond to suspected abuse | 50.78 ± 25.77 | 49.36 ± 25.37 | 52.22 ± 26.12 | -2.86 | 0.122 |
| Media exposure | Social media exposure | 47.58 ± 30.43 | 48.38 ± 28.44 | 46.77 ± 32.33 | 1.61 | 0.462 |
| Media exposure | Newspaper exposure | 26.63 ± 25.11 | 24.79 ± 23.73 | 28.50 ± 26.34 | -3.71 | 0.041 |
| Media exposure | Television exposure | 39.14 ± 27.78 | 37.73 ± 26.65 | 40.58 ± 28.86 | -2.85 | 0.156 |
| Media exposure | Online news exposure | 47.39 ± 30.27 | 48.45 ± 30.27 | 46.32 ± 30.26 | 2.13 | 0.327 |
| Media exposure | Other media exposure | 37.50 ± 28.83 | 36.42 ± 27.89 | 38.58 ± 29.75 | -2.16 | 0.301 |
| Punitive orientation | Sentences should be harsher | 76.56 ± 25.88 | 77.23 ± 24.68 | 75.88 ± 27.04 | 1.35 | 0.466 |
| Punitive orientation | Would impose harsher penalties | 75.40 ± 25.45 | 75.81 ± 24.45 | 74.99 ± 26.44 | 0.82 | 0.654 |
| Punitive orientation | Punishments are too lenient | 72.57 ± 26.48 | 72.19 ± 25.42 | 72.95 ± 27.54 | -0.77 | 0.687 |
| Punitive orientation | Sentences are too short | 66.10 ± 29.40 | 66.72 ± 28.59 | 65.48 ± 30.21 | 1.24 | 0.557 |
| Punitive orientation | Harsher punishment is better | 76.09 ± 25.67 | 76.81 ± 25.24 | 75.36 ± 26.10 | 1.45 | 0.431 |

Supplementary Table S10. Bivariate standardized scale scores by age group, residence type, and income

| **Scale** | **18–35 years** | **36–50 years** | **Over 50 years** | **p (age)** | **η² (age)** | **City** | **Village** | **Camp/Countryside** | **p (residence)** | **Low income** | **Average income** | **High income** | **p (income)** |
| --- | --- | --- | --- | --- | --- | --- | --- | --- | --- | --- | --- | --- | --- |
| Institutional trust and legitimacy | 49.55 ± 17.29 | 49.10 ± 15.67 | 52.47 ± 19.75 | 0.419 | 0.00 | 49.90 ± 17.42 | 49.68 ± 16.48 | 46.61 ± 18.43 | 0.667 | 45.28 ± 18.82 | 51.84 ± 16.29 | 47.77 ± 17.58 | <0.001 |
| Required staff expertise | 73.42 ± 19.77 | 73.23 ± 18.48 | 71.91 ± 21.12 | 0.854 | 0.00 | 73.42 ± 20.08 | 72.00 ± 18.38 | 79.13 ± 15.44 | 0.248 | 68.55 ± 22.17 | 74.02 ± 18.63 | 75.06 ± 19.57 | 0.006 |
| Perceived staff qualities | 57.75 ± 23.81 | 55.56 ± 25.04 | 46.87 ± 18.61 | 0.012 | 0.02 | 56.17 ± 24.40 | 57.44 ± 22.26 | 62.68 ± 18.41 | 0.553 | 50.77 ± 24.79 | 59.32 ± 23.06 | 53.93 ± 24.10 | 0.003 |
| Perpetrator accountability | 67.10 ± 35.96 | 68.74 ± 35.79 | 74.24 ± 32.60 | 0.329 | 0.00 | 67.90 ± 36.17 | 67.93 ± 35.34 | 66.96 ± 28.67 | 0.992 | 65.51 ± 36.23 | 67.96 ± 36.00 | 69.55 ± 34.80 | 0.610 |
| Institutional response orientation | 55.95 ± 18.56 | 57.90 ± 17.78 | 57.57 ± 18.00 | 0.476 | 0.00 | 56.63 ± 18.67 | 56.11 ± 17.36 | 55.22 ± 20.40 | 0.898 | 52.49 ± 19.81 | 57.90 ± 18.21 | 55.85 ± 17.45 | 0.009 |
| Media exposure | 39.56 ± 23.59 | 40.21 ± 21.80 | 37.41 ± 19.44 | 0.733 | 0.00 | 39.60 ± 23.52 | 39.31 ± 20.71 | 40.91 ± 28.28 | 0.953 | 38.77 ± 22.45 | 41.46 ± 23.23 | 35.25 ± 22.55 | 0.009 |
| Punitive orientation | 72.90 ± 23.84 | 74.78 ± 22.37 | 74.31 ± 20.92 | 0.663 | 0.00 | 73.70 ± 23.51 | 71.85 ± 22.66 | 75.48 ± 24.99 | 0.601 | 66.22 ± 26.08 | 74.59 ± 22.29 | 75.68 ± 22.92 | <0.001 |

Note: This table corresponds to old main-text Table 4 and should be moved to the supplementary material to reduce density of the main manuscript. Values are mean ± SD unless otherwise indicated.
